# SAG101 Forms a Ternary Complex with EDS1 and PAD4 and Is Required for Resistance Signaling against Turnip Crinkle Virus

**DOI:** 10.1371/journal.ppat.1002318

**Published:** 2011-11-03

**Authors:** Shifeng Zhu, Rae-Dong Jeong, Srivathsa C. Venugopal, Ludmila Lapchyk, DuRoy Navarre, Aardra Kachroo, Pradeep Kachroo

**Affiliations:** 1 Department of Plant Pathology, University of Kentucky, Lexington, Kentucky, United States of America; 2 U.S. Department of Agriculture–Agricultural Research Service, Washington State University, Prosser, Washington, United States of America; University of California Riverside, United States of America

## Abstract

EDS1, PAD4, and SAG101 are common regulators of plant immunity against many pathogens. EDS1 interacts with both PAD4 and SAG101 but direct interaction between PAD4 and SAG101 has not been detected, leading to the suggestion that the EDS1-PAD4 and EDS1-SAG101 complexes are distinct. We show that EDS1, PAD4, and SAG101 are present in a single complex *in planta*. While this complex is preferentially nuclear localized, it can be redirected to the cytoplasm in the presence of an extranuclear form of EDS1. PAD4 and SAG101 can in turn, regulate the subcellular localization of EDS1. We also show that the Arabidopsis genome encodes two functionally redundant isoforms of EDS1, either of which can form ternary complexes with PAD4 and SAG101. Simultaneous mutations in both *EDS1* isoforms are essential to abrogate resistance (R) protein-mediated defense against turnip crinkle virus (TCV) as well as *avrRps4* expressing *Pseudomonas syringae*. Interestingly, unlike its function as a PAD4 substitute in bacterial resistance, SAG101 is required for R-mediated resistance to TCV, thus implicating a role for the ternary complex in this defense response. However, only EDS1 is required for HRT-mediated HR to TCV, while only PAD4 is required for SA-dependent induction of HRT. Together, these results suggest that EDS1, PAD4 and SAG101 also perform independent functions in HRT-mediated resistance.

## Author Summary

Plant immunity to pathogens requires several proteins, including EDS1, PAD4, SAG101, and these are thought to act downstream of resistance protein-mediated signaling. EDS1 interacts with both PAD4 and SAG101 but no interaction has been detected between SAG101 and PAD4. We show that SAG101 interacts with PAD4 via EDS1 and that the SAG101-EDS1-PAD4 ternary complex is present in the nucleus. EDS1, which is present in the cytoplasm and nucleus, is detected preferentially in the nucleus in the presence of SAG101. The presence of PAD4 restores the cytoplasmic localization of EDS1. Conversely, the SAG101-EDS1-PAD4 ternary complex, which is detected primarily in the nucleus, is redirected to cytoplasm in the presence of an extranuclear form of EDS1. These results show that protein localization changes in relation to the subcellular localization and/or relative levels of their interacting partners. We further show that Arabidopsis plants encode two functional isoforms of EDS1. Both isoforms interact with self and each other, as well as form ternary complexes. SAG101, which is thought to serve as a substitute for PAD4, functions independently in defense signaling against turnip crinkle virus. Our results suggest that EDS1, PAD4, SAG101 function independently as well as in a ternary complex to mediate plant defense signaling.

## Introduction

One of the most studied plant defense mechanisms involves the deployment of resistance (R) proteins, which primarily provides protection against specific races of pathogens carrying corresponding avirulence (*Avr*) genes (“gene-for-gene” interactions [1]). *R* gene-mediated or race-specific immunity is induced when a strain-specific Avr protein from the pathogen associates directly/indirectly with a cognate plant R protein [2]–[4]. Induction of R-mediated responses is often accompanied by the formation of a hypersensitive response (HR) at the site of pathogen entry [5]. Although HR is considered one of the first visible manifestations of pathogen-induced host defense, whether it is the cause or consequence of resistance signaling remains unclear. Concurrent with R-mediated response, defense reactions are triggered in both local and distant parts of the plant. These include a local and systemic increase in the endogenous salicylic acid (SA) levels and the upregulation of a large set of defense genes, including those encoding pathogenesis-related (PR) proteins [6]–[7].

The SA signal transduction pathway plays a key role in plant defense signaling [8]. Arabidopsis mutants that are impaired in SA responsiveness, such as *npr1* (nonexpressor of PR [9]–[10]), or are defective in pathogen-induced SA accumulation, such as *eds1* (enhanced disease susceptibility 1 [11]), *eds5* [12], *sid2* (isochorishmate synthase [13]) and *pad4* (phytoalexin deficient 4 [14]), exhibit enhanced susceptibility to pathogen infection and show impaired *PR* gene expression. The EDS1, EDS5, PAD4, NPR1 proteins and the SA synthesizing enzyme SID2 participate in both basal and R protein-mediated defense responses [9]–[14]. EDS1 interacts with PAD4 and SAG (senescence associated gene) 101 and the combined activities of EDS1 and PAD4 proteins are required for HR formation and the restriction of pathogen growth [15]–[17]. EDS1 is thought to form two distinct complexes with PAD4 and SAG101 since direct interaction between PAD4 and SAG101 has not been detected. EDS1 and PAD4 are present in the nucleus and cytoplasm, whereas SAG101 preferentially localizes to the nucleus. A recent study suggested that both the nuclear and cytosolic fractions of EDS1 are required to complement *eds1*-conferred enhanced susceptibility [18]. SAG101 is thought to be functionally redundant with PAD4, because a mutation in *SAG101* alone does not confer bacterial susceptibility. However, *sag101 pad4* double mutant plants do exhibit significantly enhanced susceptibility in comparison to *pad4* single mutants [17], [19]. EDS1, PAD4, and SAG101 are structurally related to lipase/esterase-like proteins although lipase-like biochemical activities have not been demonstrated for EDS1 or PAD4 [11], [16], [17].

EDS1 was thought to participate in the resistance signaling mediated by toll-interleukin-nucleotide binding site-leucine rich repeat (TIR-NBS-LRR) category of R proteins [20]. However, recent results have shown that EDS1 and SA function redundantly in R-mediated signaling and this masks the requirement for EDS1 [21]. Thus, the requirement for EDS1 by R proteins previously thought to be independent of EDS1, became evident only in plants lacking the capacity to synthesize pathogen-responsive SA [21]. This includes *RPS2*, *RPP8*, and *HRT*, which encode coiled coil (CC)-NBS-LRR type R proteins and confer resistance to bacterial, oomycete and viral pathogens, respectively.

The R protein HRT confers resistance to turnip crinkle virus (TCV) and requires EDS1 and SA for resistance signaling; mutations in either EDS1 or SA synthesizing enzyme SID2 are sufficient to compromise resistance to TCV [22]. However, EDS1 and SA fulfill redundant functions in HR mediated by HRT; HR to TCV is only compromised in plants lacking EDS1 as well as SA [21]. Besides EDS1 and SA, HRT-mediated resistance also requires PAD4 and EDS5, a recessive locus *rrt* (regulates resistance to TCV), and the blue-light photoreceptors [22]-[24]. Although SA appears to function downstream of the HRT-derived recognition of TCV, it cannot confer resistance in the absence of HRT [22], [23], [25], [26]. Exogenous SA confers resistance in *HRT* background by upregulating expression of *HRT* [22]–[25]. Interestingly, the requirement for *rrt* in resistance can be overcome by increasing the levels of *HRT* via exogenous application of SA or by transgenic overexpression of *HRT* [22]–[25], [27]. HRT-mediated signaling is activated in the presence of TCV coat protein (CP) [27]–[29]. However, direct interactions between HRT and CP have not been detected.

Here, we examined the roles of EDS1, PAD4 and SAG101 in HRT-mediated signaling. We find that EDS1, but not PAD4 or SAG101, is required for CP-triggered HR in HRT expressing plants. This correlates with direct interactions between EDS1 and HRT. We also show that SAG101, which forms a ternary complex with EDS1 and PAD4, is an essential component of HRT-mediated signaling. Not only does SAG101 interact with PAD4 in the presence of EDS1, but it also induces the nuclear localization of EDS1. Conversely, the subcellular localization of the SAG101-EDS1-PAD4 ternary complex is driven by the location of EDS1. These results suggest that the inability of extranuclear EDS1 to complement *eds1-1* phenotypes might be due to the altered localization of PAD4 and SAG101. Our studies also show that the Arabidopsis genome encodes two functionally redundant EDS1 isoforms, both of which can function in the R-mediated response to TCV or *Pseudomonas syringae*.

## Results

### Two *EDS1* isoforms function redundantly in pathogen defense

Genetics analysis of F2 plants derived from crosses between resistant ecotype Di-17 and susceptible plants *eds1-1* (Ws ecotype) or *eds1-2* (L*er* ecotype) mutants showed that all *HRT eds1* plants were susceptible to TCV (Table S1, [22], [24]). In comparison, ∼25% of F2 progeny (homo/heterozygous for *HRT,* but homozygous for *rrt*) were able to resist TCV infection in control crosses between Di-17 and Col-0/Ws/L*er* [Table S1, 22]. Surprisingly, F2 progeny derived from a Di-17 x *eds1-22* (*At3 g48090;* Col-0 ecotype, [21]) cross showed normal segregation of resistant plants; 25% of *HRT* plants showed resistance (Table S1). We investigated this further and realized that previous reports had indicated the presence of two *EDS1* isoforms in the Arabidopsis ecotype Col-0 [17], [30], only one of which (encoded by *At3 g48090*, redesignated EDS1-90) has been functionally characterized [16], [17]. The other isoform (encoded by *At3 g48080*, designated EDS1-80) exhibits ∼85% amino acid (aa) identity with EDS1-90 (Figure S1A) and its transcript is induced by SA and TCV, similar to *EDS1-90* (Figures S1B, S1C). Similar to Col-0, both Di-17 and L*er* plants expressed both *EDS1-80* and *EDS1-*90 but the *EDS1-80* gene in L*er* and Di-17 contained a 28 bp deletion in the second exon (Figures S1D, S1E). This deletion would result in the expression of a truncated EDS1-80 protein comprising of only the first 162 aa instead of the 629 aa long full-length protein. Thus, L*er-eds1-2* plants would essentially be defective in both *EDS1* isoforms. Similarly, RT-PCR analysis showed that Ws and Ws-*eds1-1* genotypes express *EDS1-90*, but not *EDS1-80* (Figure S1F), suggesting that similar to L*er-eds1-*2, Ws-*eds1-1* plants are also compromised in the activities of both isoforms. These results also suggested that the presence of a functional *EDS1-90* isoform in Di-17 was sufficient for HRT-mediated resistance to TCV. To reconfirm this we isolated a T-DNA knockout (KO) mutant in *EDS1-80* in the Col-0 background (designated *eds1-80*; Figure S2A), crossed this KO line with Di-17 and analyzed segregation of resistance in the F2 plants (Table S1). Similar to Di-17 x Col-0 cross, plants of the *HRT eds1-80* genotypes segregated normally for resistance; ∼25% plants were resistant to TCV (Table S1, Figures S2B, S2C). Genetic analysis based on *EDS1-90* and *EDS1-80* KO mutants suggested that either of the *EDS1* isoforms can mediate HRT-mediated resistance to TCV.

We tested this further by evaluating the response of another *R* gene *RPS4,* which mediates resistance to *Pseudomonas syringae* expressing *AvrRps4* (Figure S3) and is known to require EDS1. Unlike wild-type Ws plants, inoculation of *avrRps4* bacteria induced prominent chlorosis and cell death in Ws-*eds1-1* plants. The Col-0-*eds1-80* and Col-0-*eds1-90* plants on the other hand showed a similar response as wild-type Col-0 plants (Figures S3A, S3B). Similarly, pathogen inoculation induced SA and *PR-1* levels in wild-type and *eds1-80* or *eds1-90* plants, but not in *eds1-1* (Figures S3C, S3D). The *eds1-80* or *eds1-90* plants supported similar levels of bacterial growth as wild-type plants, which were ∼40-fold lower than that of the *eds1-1* plants (Figure S3E). Together, these results show that single mutations in *EDS1-80* or *EDS1-90* in Col-0 background were insufficient to compromise *RPS4*-mediated resistance against *avrRps4* bacteria.

**Figure 1:**
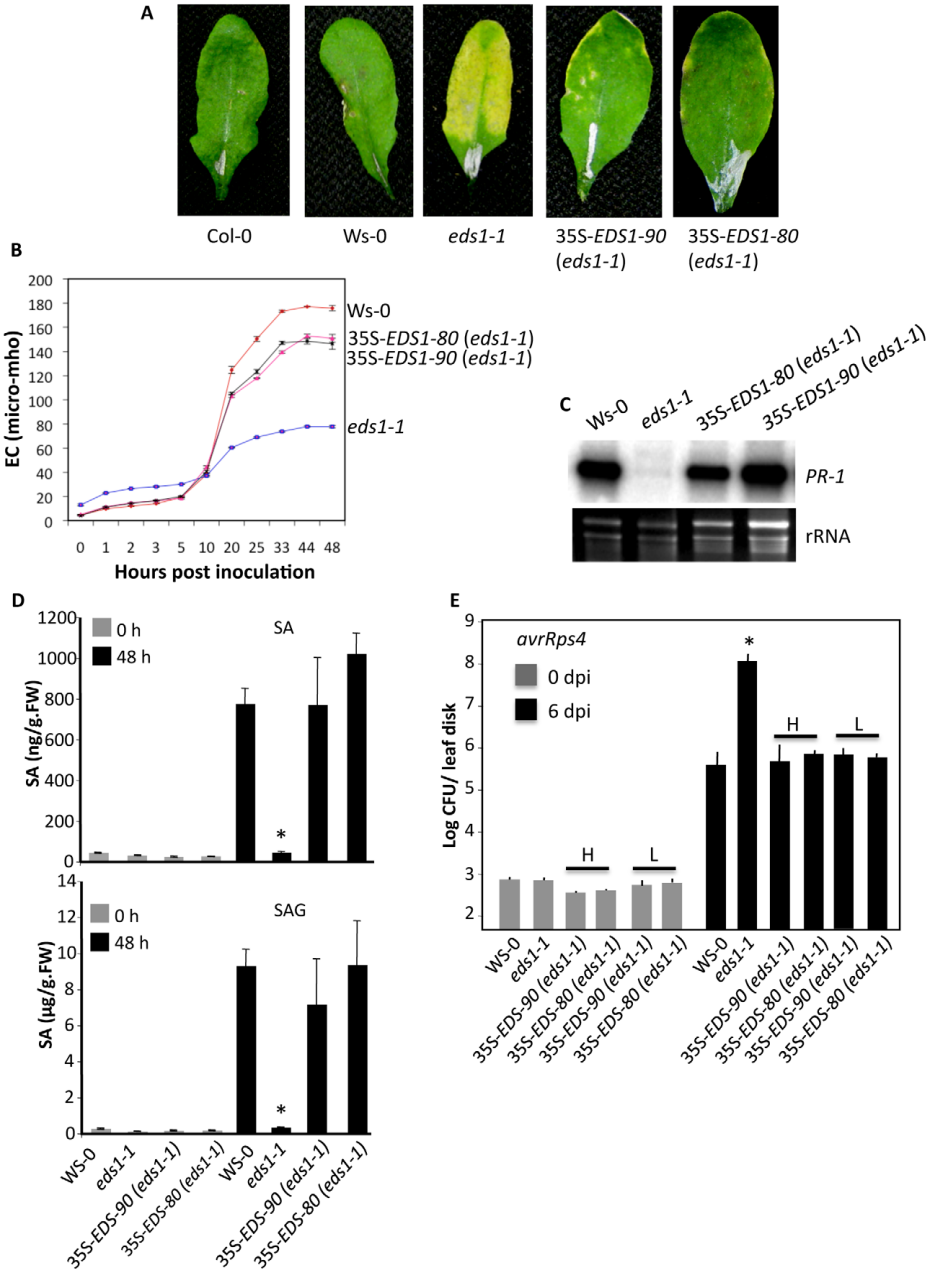
The *EDS1-80* or *EDS1-90* genes complement the compromised defense phenotypes in *eds1-1* plants. (**A**) Photograph showing phenotypes produced upon infiltration of 10^5^ colony forming units (CFU)/ml of *avrRps4 Pseudomonas syringae*. The leaves were photographed at 6 days post inoculation (dpi). (**B**) Electrolyte leakage in *avrRps4* inoculated genotypes. Error bars represent SD (n = 6). (**C**) *PR-1* gene expression in *avrRps4* inoculated plants. Leaves were sampled at 2 dpi. Ethidium bromide staining of rRNA was used as a loading control. (**D**) Salicylic acid (SA) and SA glucoside (SAG) levels in indicated genotypes at 0 and 48 h post inoculation with *avrRps4*. Asterisks indicate data statistically significant from wt Col-0 ecotype (P<0.05, n = 3). The error bars indicate SD. (**E**) Growth of *avrRps4* bacteria on indicated genotypes. The error bars indicate SD. Asterisks indicate data statistically significant from wt (Col-0, P<0.05 n = 4). H and L indicate transgenic plants expressing high and low levels of *EDS1*, respectively (see Figures S4A, S4B).

To determine if *EDS1-80* and *EDS1-90* encoded functional proteins, corresponding to their orthologs in Ws and L*er* ecotypes, we tested their ability to complement *eds1-1* phenotypes. The *EDS1-80* and *EDS1-90* isoforms were expressed under the 35S promoter in the *eds1-1* background (Figures S4A, S4B) and the T2 plants obtained from four independent lines expressing low or high *EDS1* transcripts were analyzed for resistance to *avrRps4* bacteria. Typical chlorosis and cell death phenotypes associated with *avrRps4* infection on *eds1-1* plants were not evident in plants expressing *EDS1-80* or *EDS1-90*, regardless of their transcript levels (Figures 1A, S4C). Concurrently, these plants showed wt-like levels of ion-leakage (Figure 1B), *PR-1* expression (Figure 1C), and SA levels (Figure 1D) in response to *avrRps4* inoculation. The *eds1-1* plants expressing *EDS1-80* or *EDS1-90* also supported wt-like growth of *avrRps4* bacteria (Figure 1E). Together, these results suggest that both *EDS1-80* and *EDS1-90* encode functional proteins and expression of either gene complements the enhanced disease susceptibility phenotype in *eds1-1* plants. Together, these results suggest that the two *EDS1* isoforms likely function redundantly and that simultaneous mutations in both *EDS1* isoforms are required to compromise *HRT*-mediated resistance to TCV.

### EDS1 interacts with HRT and promotes HRT-CP-mediated HR

**Figure 2:**
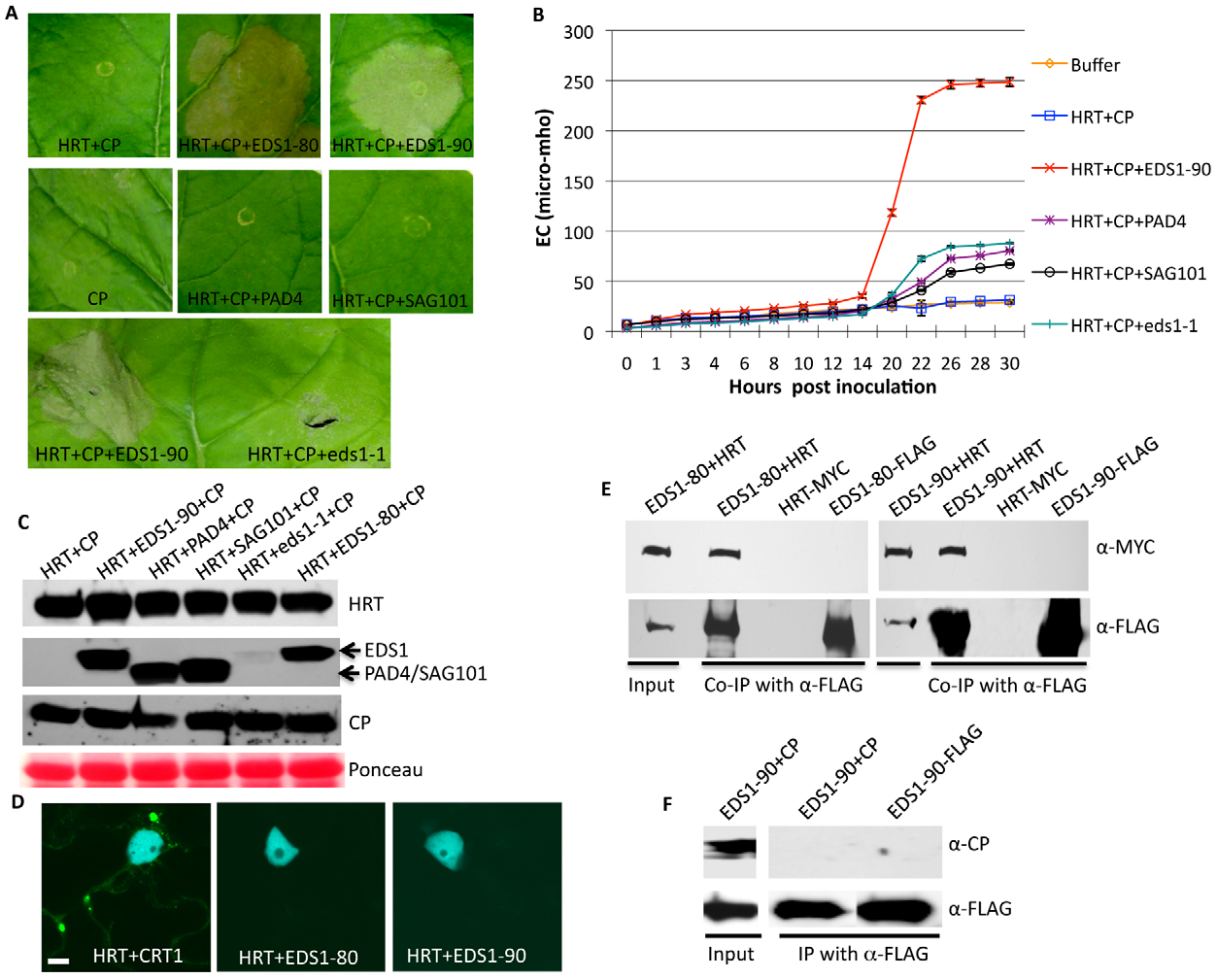
EDS1 interacts with HRT and promotes HRT-mediated cell death. (**A**) Visual phenotype of *N. benthamiana* leaves expressing indicated proteins. Agroinfiltration was used to express HRT, CP, EDS1-80, EDS1-90, and eds1-1 proteins and the leaves were photographed at 3 days post treatment. (**B**) Electrolyte leakage in *N. benthamiana* leaves shown in A. Error bars represent SD (n = 6). (**C**) Immunoblot showing levels of HRT, EDS1, PAD4, SAG101 and CP in plants shown in A. Ponceau-S staining of the Western blot was used as the loading control. (**D**) Confocal micrographs showing bimolecular fluorescence complementation (BiFC) for indicated proteins. Agroinfiltration was used to express proteins in transgenic *N. benthamiana* plants expressing the nuclear marker CFP-H2B (Scale bar, 10 µM). The micrographs shown are CFP and YFP overlay images. (**E**) Co-immunoprecipitation (IP) of HRT-MYC with EDS1-80 or EDS1-90 FLAG. *N. benthamiana* plants were agroinfiltrated and total extracts (input) were immunoprecipitated with α-FLAG and analyzed with α-MYC or α-FLAG antibodies. (**F**) Co-IP of CP with EDS1-90-FLAG. *N. benthamiana* plants were agroinfiltrated and total extracts (input) were immunoprecipitated with α-FLAG and analyzed with α-CP or α-FLAG antibodies.

To determine if EDS1 was required for the activation of HRT-mediated signaling we developed a transient system based on reconstitution of HR in *Nicotiana benthamiana*. This was essential since EDS1 and SA act redundantly to regulate HRT-mediated signaling in Arabidopsis [21], thereby rendering it difficult to test the function of EDS1 alone. This assay was facilitated by the fact that co-infiltration of HRT and its cognate avirulence effector CP induced a delayed and weak HR in *N. benthamiana*. Thus, any factor participating in the activation of HRT-mediated signaling should promote HR formation. Interestingly, co-infiltration of EDS1-80 or EDS1-90 together with HRT and CP promoted HR formation (Figure 2A), suggesting that both EDS1 isoforms likely facilitate the recognition of CP. This was further confirmed by assaying ion-leakage (Figure 2B). Unlike EDS1, co-infiltration of the eds1-1 mutant protein, SAG101 or PAD4 did not induce a strong HR in the presence of HRT and CP (Figures 2A, 2B). HRT and CP were expressed at comparable levels in the presence or absence of SAG101 and PAD4, suggesting that lack of HR in the HRT+CP+SAG101/PAD4 plants was not due to insufficient levels of HRT and/or CP (Figure 2C). Likewise, expression levels of SAG101 and PAD4 were similar to EDS1. Notably, as in Arabidopsis [17], eds1-1 protein was unstable and accumulated to very low levels in *N. benthamiana* (Figure 2C).

To determine if EDS1 promoted HRT+CP-dependent HR via interactions with HRT and/or CP, we carried out bimolecular fluorescence complementation (BiFC) assays (Figure 2D). As expected, HRT associated with its interacting partner CRT1 [21], [31], but no interaction was detected between either EDS1 isoforms and HRT or CP (Figure 2D). However, co-immunoprecipitation (IP) assays showed that EDS1 interacted with HRT, but not CP (Figures 2E, 2F). Neither HRT nor EDS1 interacted with GST (data not shown). Consistent with their ability to promote HR formation, both EDS1 isoforms associated with HRT in co-IP assays. Interaction between EDS1 and HRT was further verified by expressing these proteins under their native promoters in *N. benthamiana* plants (Figure S5A) as well as in Arabidopsis protoplasts (Figure S5B). Notably, EDS1 accumulated to similar levels when expressed under the 35S or its native promoter (Figure S5C). In comparison, HRT accumulated to higher levels when expressed under its native promoters, compared to 35S (Figure S5D). These results argue that interaction between HRT and EDS1 was not due to overexpression of these proteins. Together these results suggest that HRT associates with EDS1, albeit indirectly, and this likely facilitates the CP-triggered induction of HR in the presence of HRT.

### EDS1-80 interacts with PAD4 and SAG101

**Figure 3:**
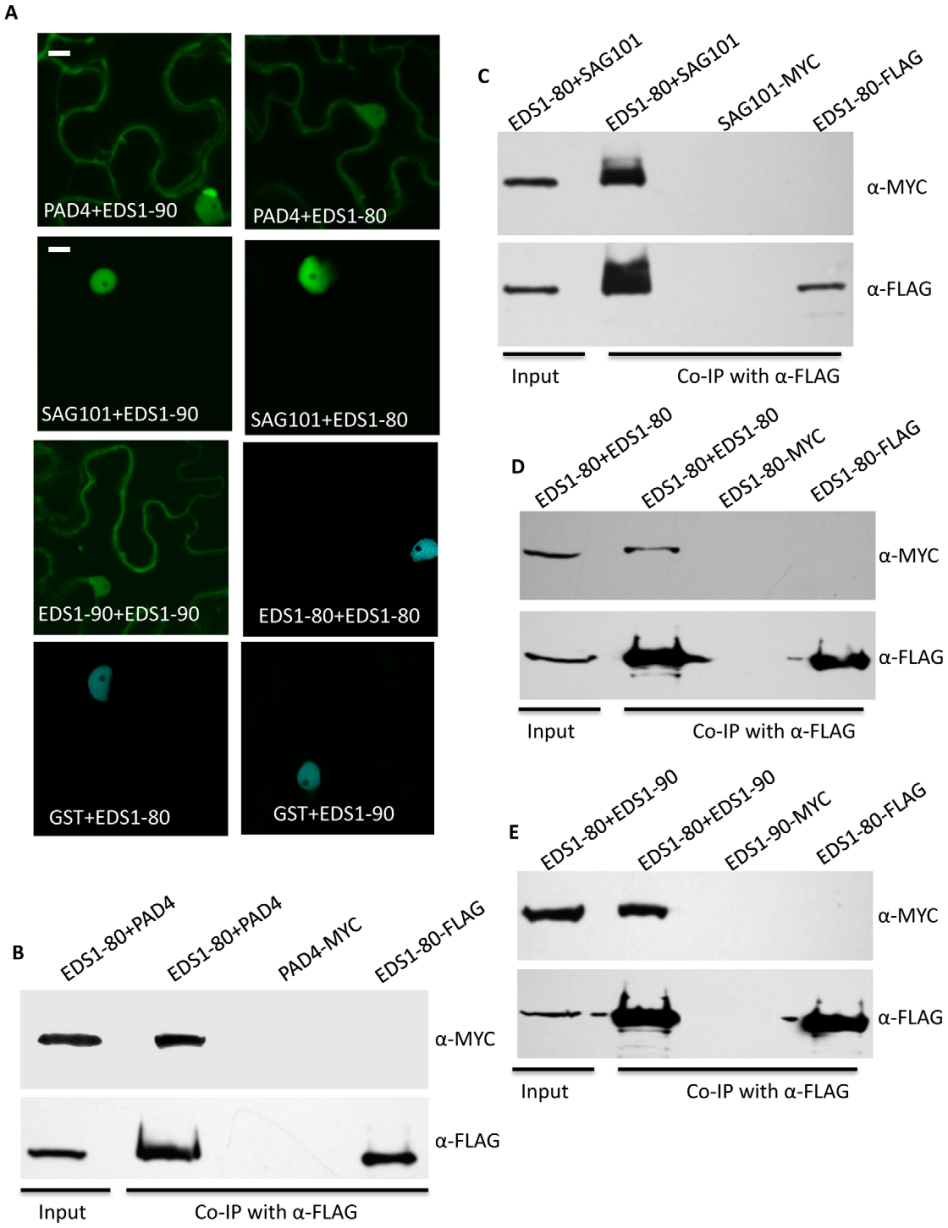
EDS1-80 interacts with SAG101, PAD4 and EDS1-90. (**A**) Confocal micrographs showing BiFC for indicated proteins. Agroinfiltration was used to express protein in transgenic *N. benthamiana* plants expressing the nuclear marker CFP-H2B (Scale bar, 10 µM). The micrographs shown are CFP and YFP overlay images. (**B–E)** Co-IP of PAD4-MYC (**B**) SAG101-MYC (**C**) EDS1-80-MYC (**D**) and EDS1-90-MYC proteins (**E**) with EDS1-80-FLAG. *N. benthamiana* plants were agroinfiltrated and total extracts (input) were immunoprecipitated with α-FLAG and analyzed with α-MYC or α-FLAG antibodies.

In view of the functional redundancy between EDS1-80 and 90 and their association with HRT, it was important to determine if EDS1-80 was capable of forming a complex with PAD4 and SAG101. Indeed, similar to EDS1-90, EDS1-80 interacted with both PAD4 and SAG101 but not GST; the EDS1-80-PAD4 interaction was detected in both the periphery and nucleus of plant cells (Figure 3A). In comparison, the EDS1-80-SAG101 complex was primarily seen in the nucleus (Figure 3A). Co-IP assays further confirmed results obtained in the BiFC (Figures 3B, 3C). Since EDS1 and PAD4 are well known to regulate pathogen-induced accumulation of SA [11], [14], we next tested if SA altered the EDS1-80-PAD4 or EDS1-80-SAG101 interactions. No obvious differences in the intensity or site of interactions were noticed (data not shown), suggesting that increased SA might not alter these interactions. Unlike EDS1-90, the EDS1-80 isoform did not interact with itself or with EDS1-90 in BiFC assays (Figures 3A, S6A). In contrast, IP assays detected EDS1-80 interaction with itself and EDS1-90 (Figures 3D, 3E). This suggests that the homo and heterodimerization of EDS1-80 and EDS1-90 was likely indirect.

**Figure 4:**
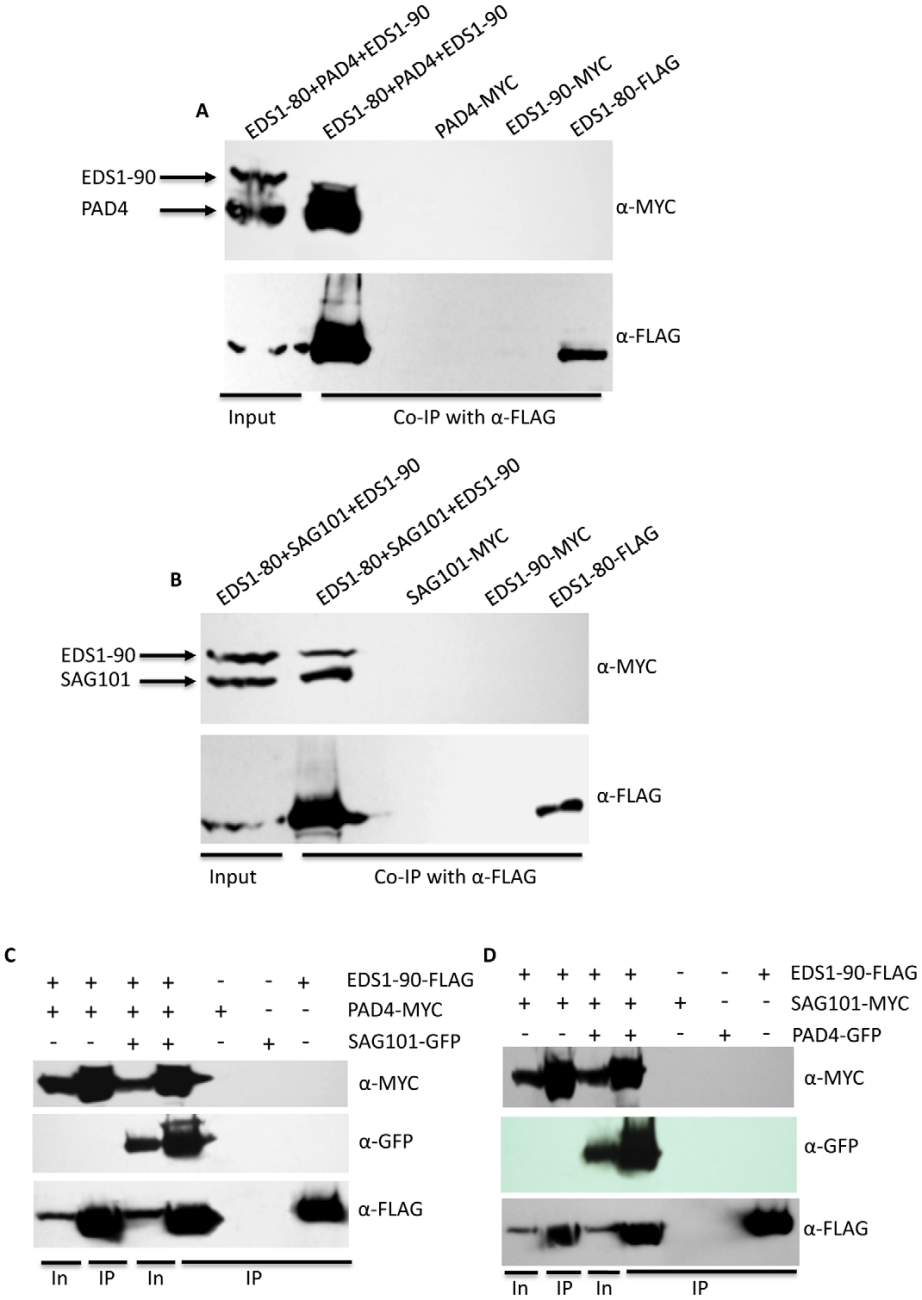
EDS1-80 shows higher affinity for PAD4 and SAG101 in comparison to EDS1-90. (**A**) Co-IP of EDS1-90-MYC and PAD4-MYC with EDS1-80-FLAG. *N. benthamiana* plants were agroinfiltrated and total extracts (input) and immunoprecipitated proteins were analyzed with α-MYC or α-FLAG. (**B**) Co-IP of EDS1-90-MYC and SAG101-MYC with EDS1-80-FLAG. *N. benthamiana* plants were agroinfiltrated and total extracts (input) and immunoprecipitated proteins were analyzed with α-MYC or α-FLAG. (**C**) Co-IP of PAD4-MYC with EDS1-90-FLAG in the presence or absence of SAG101-GFP. *N. benthamiana* plants were agroinfiltrated and total extracts (input) and immunoprecipitated proteins were analyzed with α-MYC, α-GFP or α-FLAG. (**D**) Co-IP of SAG101-MYC with EDS1-90-FLAG in the presence or absence of PAD4-GFP. *N. benthamiana* plants were agroinfiltrated and total extracts (input) and immunoprecipitated proteins were analyzed with α-MYC, α-GFP or α-FLAG. Band intensities in C and D were quantified using an ImageQuant program.

We next tested whether the presence of PAD4 or SAG101 affected the formation of the EDS1-80-90 heterodimer. EDS1-80 and EDS1-90 were co-expressed with PAD4 or SAG101, and immunoprecipitates were assayed for the EDS1-90, PAD4 or SAG101 proteins. Interestingly, EDS1-80 preferentially bound PAD4 in the presence of EDS1-90 (Figures 4A, S6B). EDS1-80 also showed slightly more affinity for SAG101 over EDS1-90 (Figure 4B). We next compared the relative affinities of EDS1-90 for PAD4 and SAG101. Similar levels of EDS1-PAD4 complex were detected in the absence or presence of SAG101 (Figure 4C). Similarly, levels of the EDS1-SAG101 complex did not alter significantly in the presence or absence of PAD4 (Figure 4D). We next assayed interaction of SAG101 and PAD4 with the lipase (LP; N-t 351 aa) and EDS1-PAD4-like (EP; C-t 351–623) domains of EDS1. Both SAG101 and PAD4 interacted with the LP, but not the EP, domain of EDS1 (Figure S6C). Together, these results suggest that SAG101 and PAD4 likely interact with different residues within the LP domain of EDS1 and therefore do not compete for binding with EDS1.

### EDS1 preferentially localizes to the nucleus in the presence of SAG101

**Figure 5:**
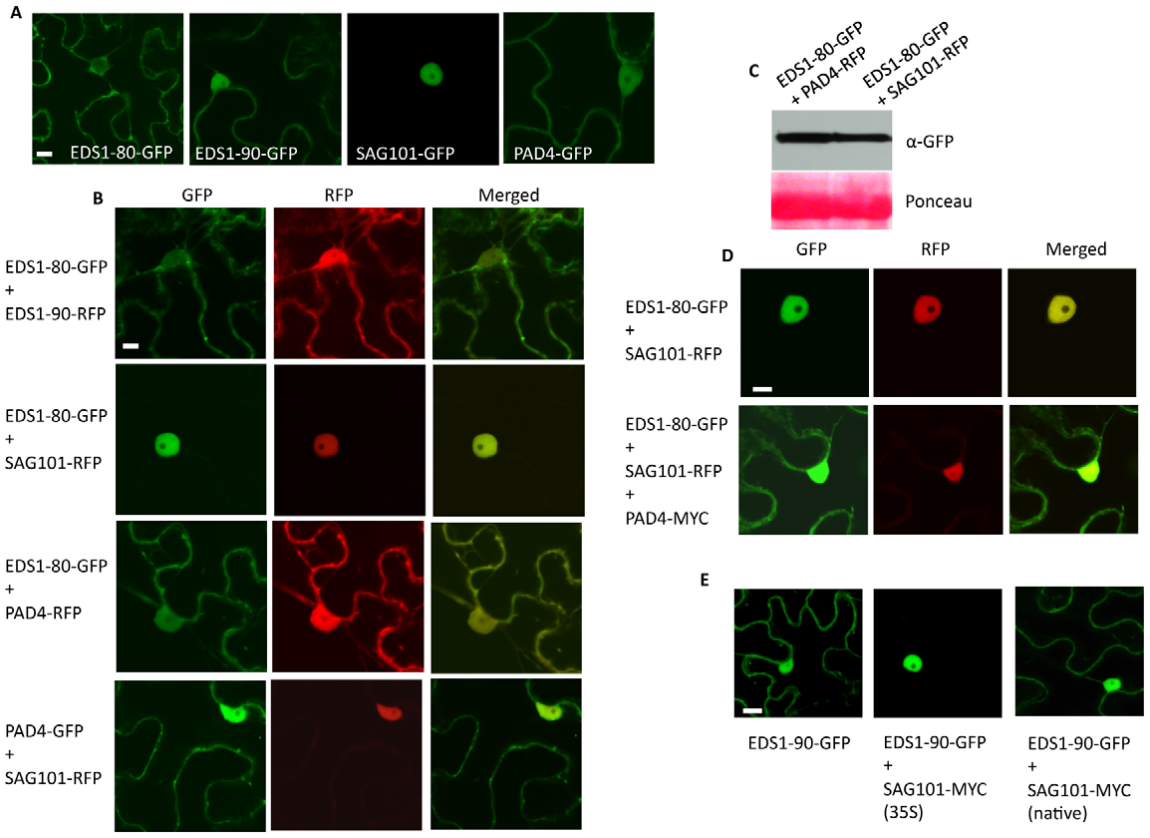
EDS1 preferentially localizes to the nucleus in the presence of SAG101. (**A**) Confocal micrographs showing localization of EDS1-80, EDS1-90, SAG101 and PAD4 proteins (Scale bar, 10 µM). (**B**) Confocal micrographs showing localization of the indicated proteins pairs (Scale bar, 10 µM). (**C**) Immunoblot showing levels of EDS1-80-GFP in plants co-infiltrated with PAD4-RFP or SAG101-RFP. Ponceau-S staining of the Western blot was used as loading control. (**D**) Confocal micrograph showing localization of proteins in *N. benthamiana* expressing EDS1-80 with SAG101 or both SAG101 and PAD4 (Scale bar, 10 µM). Expression of PAD4 was confirmed by immunoblot analysis (data not shown). (**E**) Confocal micrograph showing localization of EDS1 in *N. benthamiana* in the absence or presence of SAG101 expressed under 35S or native promoters (Scale bar, 10 µM).

We noticed in our BiFC assays that the EDS1-SAG101 interaction occurred primarily in the nucleus (Figure 3A), even though EDS1-80 or 90 were present in both the cytosol and the nucleus (Figure 5A). We tested whether SAG101 influenced the subcellular localization of EDS1 and/or PAD4. Coexpression of EDS1-GFP with PAD4-RFP did not alter the localization of either protein; EDS1-80, EDS1-90 and PAD4 localized to the nucleus and periphery of the cell, similar to when expressed individually (Figures 5A, 5B, S7A). Similarly, coexpression of PAD4-GFP and SAG101-RFP did not alter the localization of either protein (Figure 5B). However, co-expression of EDS1-GFP with SAG101-RFP or SAG101-MYC altered the localization of EDS1, but not SAG101; in the presence of SAG101, EDS1 was preferentially detected in the nucleus (Figures 5B, S7A, S7B). This SAG101 triggered nuclear localization of EDS1-80 and EDS1-90 was not due to increased expression of EDS1 in the presence of SAG101 (Figure 5C). This result is in agreement with the previous report where co-localization of EDS1 and SAG101 was tested in wild-type Arabidopsis [17]. Interestingly, coexpression of PAD4-MYC or PAD4-Cerulean together with EDS1-GFP and SAG101-RFP retained some portion of EDS1 in the cytosol (Figures 5D, S7C). Notably, nuclear-cytoplasmic localization of EDS1 was only observed when PAD4 was coexpressed with EDS1 and SAG101. EDS1 remained preferentially in the nucleus when PAD4 was expressed 24 or 48 h after EDS1 and SAG101 (data not shown). This suggested that, rather than relocalizing EDS1, PAD4 merely retained it in the cytosol. This further suggested that EDS1 might be retained inside the nucleus in the presence of SAG101, although the nuclear localization of EDS1 was not dependent on SAG101 (data not shown). Unlike PAD4, SAG101 accumulated to higher levels when expressed under 35S, compared to its native promoter (Figures S7D, S7E). Thus, it was possible that the nuclear relocalization of EDS1 was dependent on the levels of SAG101. Indeed, when expressed under its native promoter, SAG101 was unable to relocalize all the cytosolic EDS1 into the nucleus (Figure 5E). These results, together with the observation that EDS1 and PAD4 levels change during pathogen infection [16], suggest that relative levels of EDS1, PAD4 and SAG101 might regulate distribution and/or localization of these proteins in response to pathogen stimulus.

**Figure 6:**
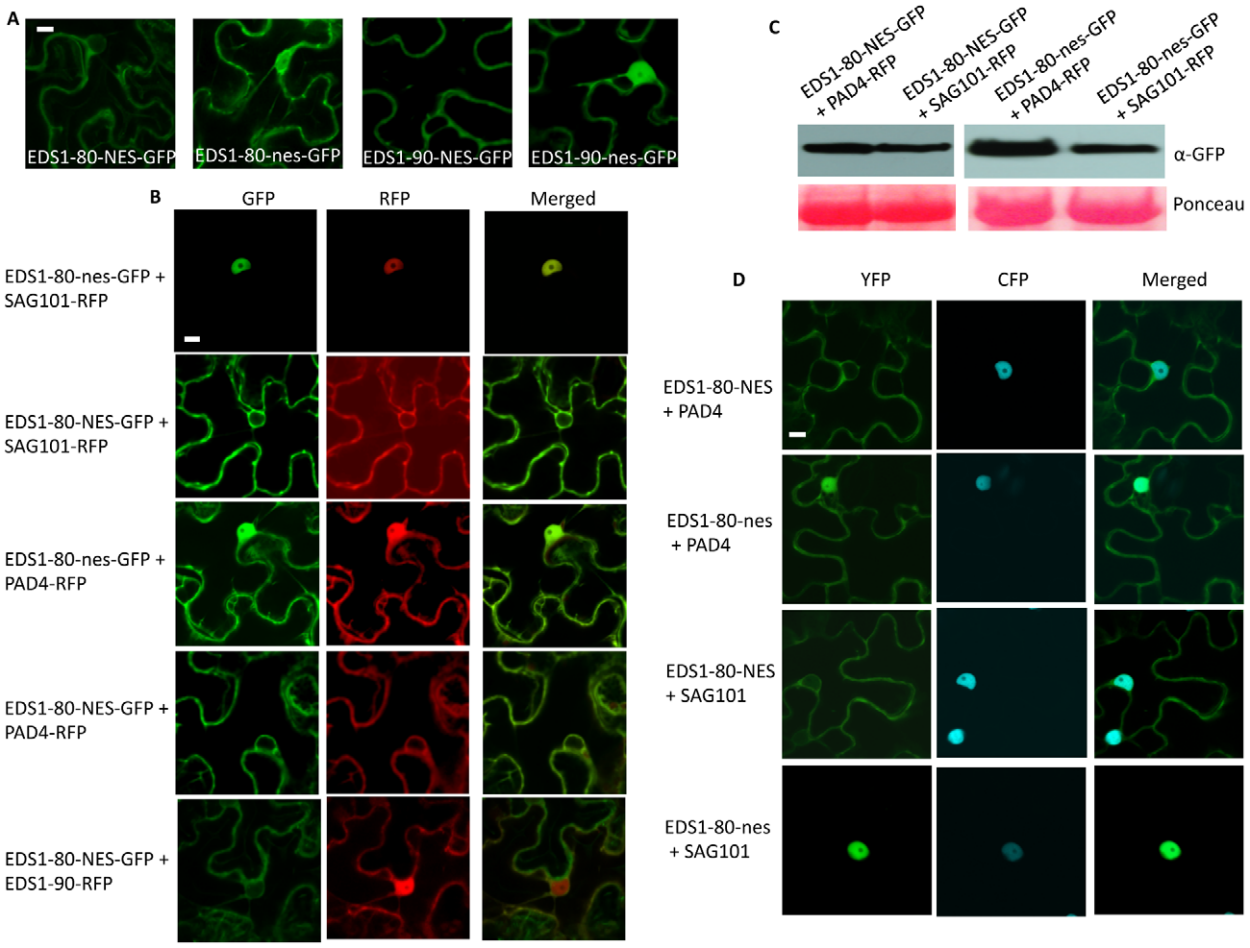
EDS1-NES exhibits extranuclear interaction with SAG101. (**A**) Confocal micrographs showing localization of EDS1-80 and EDS1-90 proteins tagged with functional (NES) or mutant (nes) nuclear export signals (Scale bar, 10 µM). (**B**) Confocal micrographs showing localization of indicated proteins co-expressed in pairs (Scale bar, 10 µM). (**C**) Immunoblot showing levels of EDS1-80-NES-GFP and EDS1-80-nes-GFP in plants co-infiltrated with PAD4-RFP or SAG101-RFP. Ponceau-S staining of the Western blot was used as loading control. (**D**) Confocal micrographs showing BiFC for indicated proteins. Agroinfiltration was used to express protein in transgenic *N. benthamiana* plants expressing the nuclear marker CFP-H2B (Scale bar, 10 µM).

To further confirm that the SAG101 triggered nuclear relocalization of EDS1 was a specific phenotype, we tagged EDS1 with a nuclear export signal (NES), or its mutant derivative (nes) [18]. As expected, both EDS1-80-NES and EDS1-90-NES were preferentially detected outside the nucleus while EDS1-80-nes and EDS1-90-nes localized like wild-type EDS1 (Figure 6A). Coexpression experiments showed that only EDS1-nes, but not EDS1-NES, relocalized to the nucleus in the presence of SAG101 (Figure 6B). Coexpression with PAD4 did not alter the localization of either form of EDS1. However, nuclear localization of PAD4 was affected by the presence of EDS1-NES, but not EDS1-nes (Figure 6B). Similar levels of EDS1-80-NES/nes protein in plants infiltrated with SAG101-RFP or PAD4-RFP suggested that localization of EDS1 was not associated with levels of protein expression (Figure 6C). Furthermore, EDS1-80-NES or EDS1-90-NES failed to induce the nuclear exclusions of RFP-tagged EDS1-90 (Figures 6B, S7D), suggesting that EDS1-NES-dependent extranuclear localization of SAG101 and PAD4 was a specific phenotype.

We next evaluated the interaction of PAD4 and SAG101 with EDS1-NES or EDS1-nes. As expected, EDS1-nes behaved similar to EDS1 (Figures 3A, 6D, S8). Notably, although EDS1-NES associated with both PAD4 and SAG101, these interactions occurred preferentially outside the nucleus (Figures 6D, S8). This was particularly evident in the case of SAG101, since the EDS1-SAG101 complex is normally located inside the nucleus. Together, these results suggest that the selective retention of EDS1 in a subcellular compartment can drive the localization of the EDS1-PAD4 and EDS1-SAG101 complexes. The fact that EDS1 can induce the cytosolic relocalization of SAG101 further suggests that, the previously reported inability of *EDS1-NES* to fully complement *eds1-1* phenotypes might be due to the altered/mis-localization of PAD4 and/or SAG101, rather than the absence of EDS1 in the nucleus [18].

**Figure 7:**
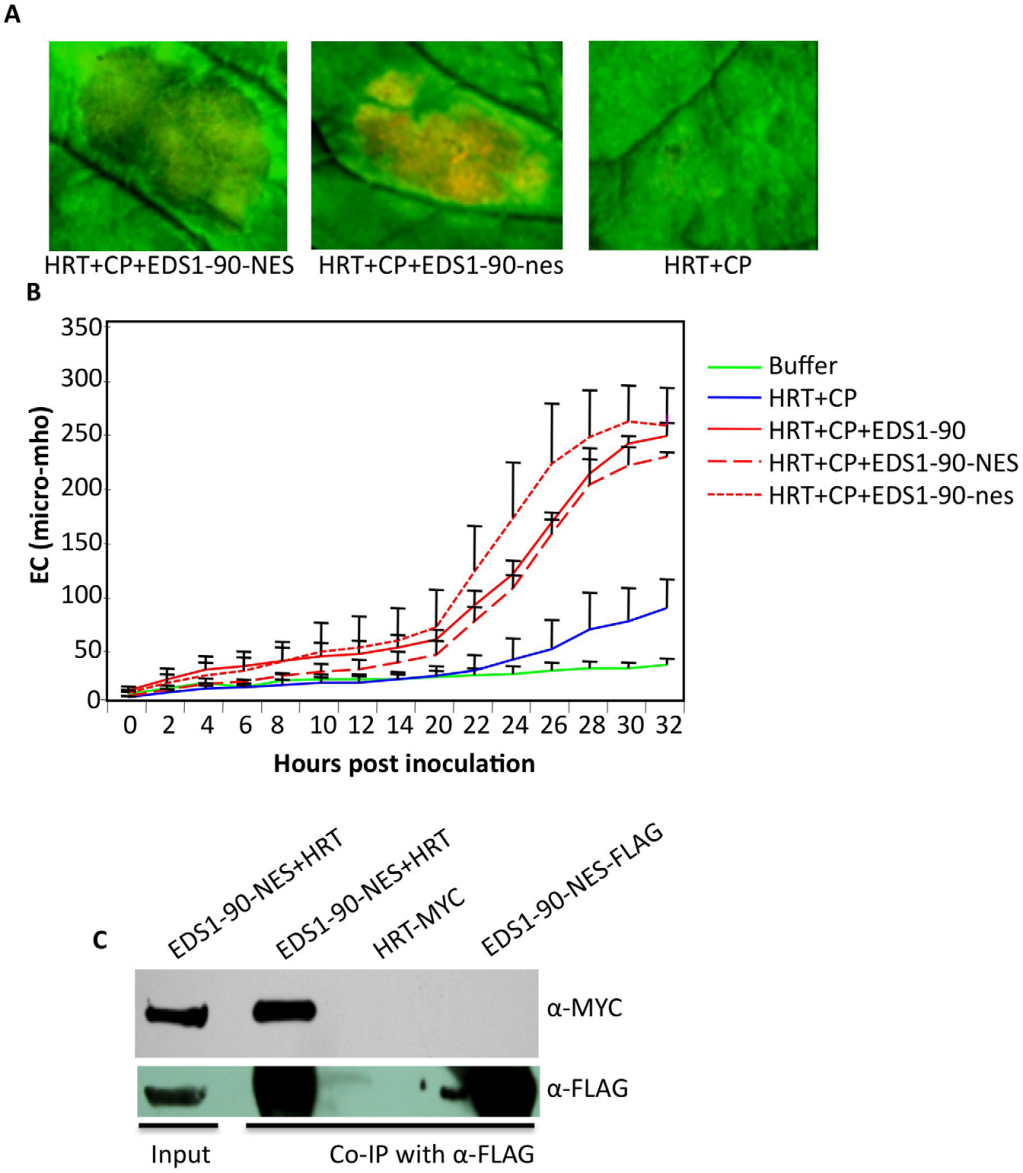
EDS1-NES interacts with HRT and promotes HRT-mediated cell death. (**A**) Visual phenotype of *N. benthamiana* leaves expressing indicated proteins. Agroinfiltration was used to express HRT, CP, EDS1-90-NES and EDS1-90-nes, proteins and the leaves were photographed at 3 days post treatment. (**B**) Electrolyte leakage in *N. benthamiana* leaves shown in A. Error bars represent SD (n = 5). (**C**) Co- IP of HRT-MYC with EDS1-90-NES-FLAG. *N. benthamiana* plants were agroinfiltrated and total extracts (input) were immunoprecipitated with α-FLAG and analyzed with α-MYC or α-FLAG antibodies.

To determine if altered localization of EDS1-NES affected its ability to promote HRT-CP triggered cell death phenotype, we monitored visual phenotypes and ion-leakage in *N. benthamiana* plants infiltrated with HRT+CP+EDS1-NES. No significant difference was noticed in HRT+CP-mediated cell death phenotype induced in the presence of EDS1, EDS1-NES or EDS1-nes (Figures 7A, 7B). This further correlated with positive interaction seen between EDS1-NES and HRT proteins (Figure 7C). Together, these results suggested that the extranuclear retention of EDS1, and by extension that of PAD4 and SAG101, do not suppress HRT+CP-mediated HR.

### EDS1, PAD4 and SAG101 exist in a single complex *in planta*

**Figure 8:**
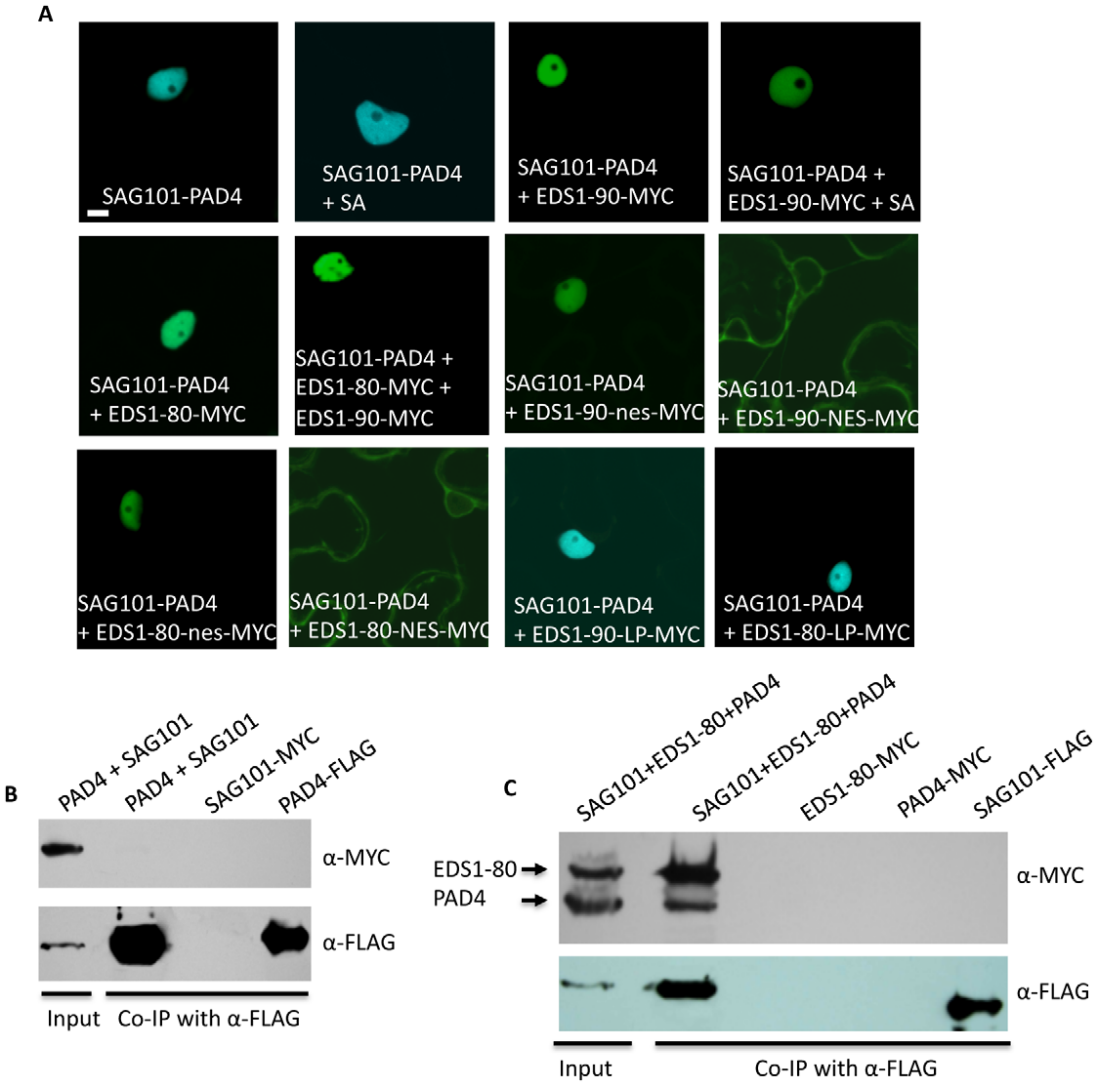
EDS1 facilitates the interaction between SAG101 and PAD4. (**A**) Confocal micrographs showing BiFC for indicated proteins. Agroinfiltration was used to express protein in transgenic *N. benthamiana* plants expressing the nuclear marker CFP-H2B (Scale bar, 10 µM). (**B**) Co-IP assay of PAD4 and SAG101 interaction. *N. benthamiana* plants were agroinfiltrated and total extracts (input) and immunoprecipitated proteins were analyzed with α-MYC or α-FLAG. (**C**) Co-IP of PAD4-MYC and EDS1-80-MYC with SAG101-FLAG. *N. benthamiana* plants were agroinfiltrated and total extracts (input) and immunoprecipitated proteins were analyzed with α-MYC or α-FLAG.

The ability of EDS1, PAD4, SAG101 proteins to relocalize their interacting partners suggested that these proteins might be present in a ternary complex. However, consistent with earlier observations [17], we were unable to detect interactions between SAG101 and PAD4 in BiFC or co-IP assays (Figures 8A, 8B). We considered the possibility that factors present only during induced defense might be required for the PAD4-SAG101 association, if any. Since both EDS1 and PAD4 are known to regulate SA levels and because exogenous SA can induce reducing conditions required for relocating proteins [32], we tested binding between SAG101 and PAD4 in plants pretreated with SA. No SAG101-PAD4 interaction was detected in SA pretreated plants (Figure 8A). Another possibility was that SAG101-PAD4 interacted via a third protein, possibly EDS1, since both SAG101 and PAD4 interacted with EDS1. We tested the SAG101-PAD4 interaction in the presence of EDS1-80 or EDS1-90 (Figure 8A). Indeed, SAG101 and PAD4 associated with each other in the presence of either EDS1 isoform. This was further confirmed using co-IP assays (Figure 8C). Increased nuclear fluorescence in the BiFC assays suggested that a majority of the SAG101-EDS1-PAD4 complex was present in the nucleus. Pretreatment with SA did not alter formation or localization of the SAG101-EDS1-PAD4 complex. Interestingly, full length EDS1 was required for SAG101-EDS1-PAD4 complex formation, even though EDS1-LP domain alone was sufficient for interaction with SAG101 or PAD4 (Figure 8A). We next assayed the interaction between SAG101 and PAD4 in the presence of EDS1-NES and EDS1-nes, which were expressed as MYC tagged proteins (Figure S9). Surprisingly, in the presence of EDS1-NES, the SAG101-PAD4 interaction was preferentially detected outside the nucleus (Figure 8A), suggesting that the subcellular location of EDS1 might drive the localization of the SAG101-EDS1-PAD4 complex.

### SAG101 is essential for HRT-mediated resistance

**Figure 9:**
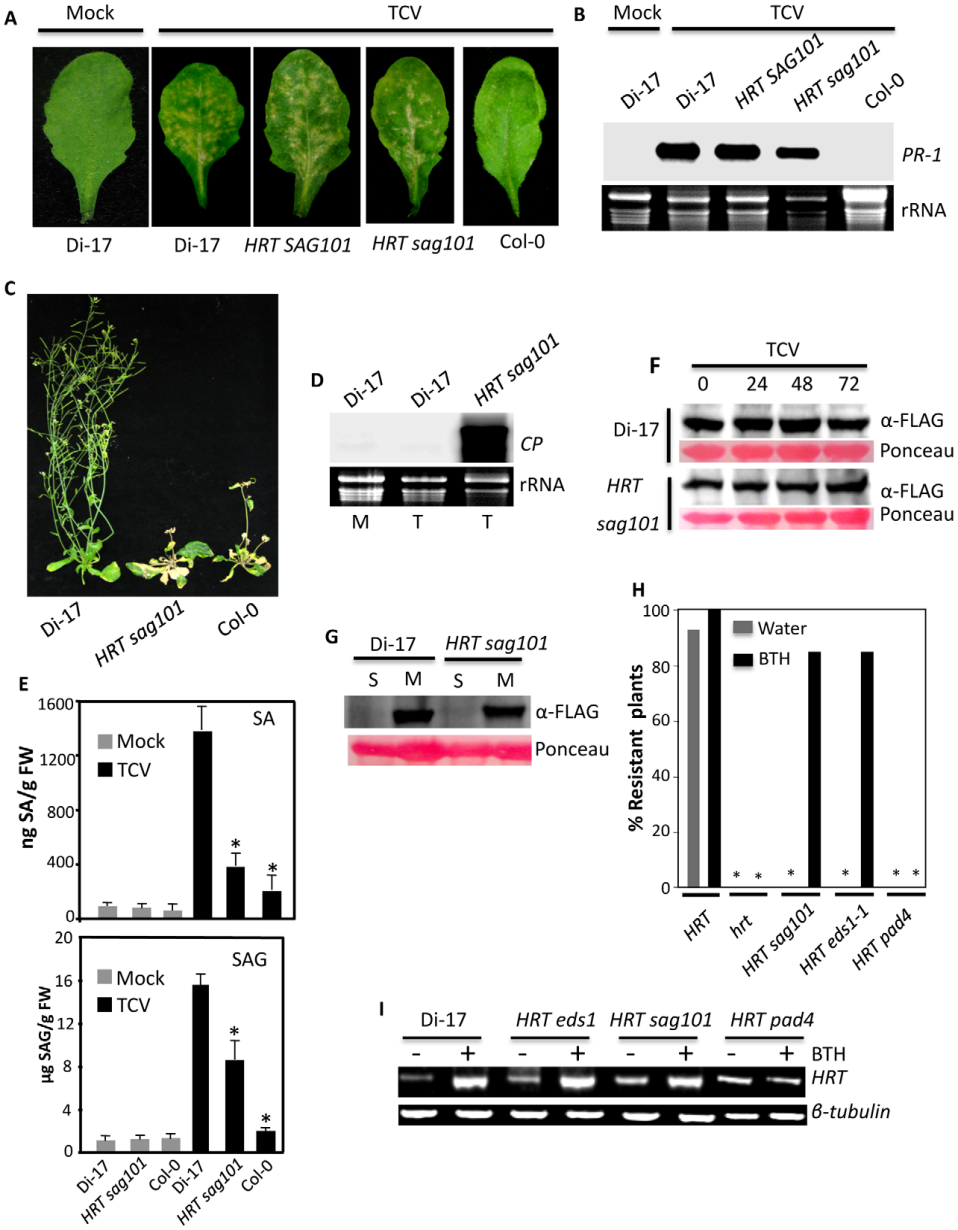
SAG101 is required for HRT-mediated resistance to TCV. (**A**) HR formation in indicated genotypes at 72 h post inoculation with TCV. (**B**) Expression of *PR-1* gene in indicated genotypes after mock- or TCV-inoculation. Total RNA was extracted from inoculated leaves at 3 dpi. Ethidium bromide staining of rRNA was used as the loading control. (**C**) Typical morphological phenotypes of TCV inoculated Di-17, *HRT sag101* and Col-0 plants. Plants were photographed at 14 dpi. (**D**) Transcript levels of TCV-CP in the distal leaves of mock (M) or TCV (T) inoculated plants. Ethidium bromide staining of rRNA was used as the loading control. (**E**) SA and SAG levels in indicated genotypes at 72 h post inoculation with buffer (Mock) or TCV. Asterisks indicate data statistically significant from wt Di-17 (P<0.05, n = 6). The error bars indicate SD. (**F**) Western blot showing HRT-FLAG levels in indicated genotypes 0–72 h post inoculation with TCV. Ponceau-S staining of the Western blot was used as the loading control. (**G**) HRT-FLAG levels in soluble (S) and membrane (M) fractions extracted from Di-17 and *HRT sag101* plants. Ponceau-S staining of the Western blot was used as the loading control. (**H**) Percentage resistant plants obtained in indicated genotypes that were treated with water (gray bars) or BTH (black bars) for 48 h before TCV inoculation. Approximately 40–50 plants were inoculated in four separate experiments and analyzed for resistance phenotype. Asterisks denote 100% susceptibility. (**I**) RT-PCR analysis showing *HRT* transcript levels in indicated genotypes treated with water or BTH for 48 h prior to sampling. The level of *β-tubulin* was used as an internal control to normalize the amount of cDNA template.

The fact that SAG101 forms a ternary complex with EDS1 and PAD4, and that it can drive the nuclear localization of EDS1 is inconsistent with a proposed redundant role for SAG101 in plant defense [17], [19]. We therefore investigated the requirement for *SAG101* in *HRT*-mediated signaling. We crossed Di-17 plants with *sag101* and analyzed HR and resistance in the F_2_ population. Approximately 75% of the plants showed HR to TCV, regardless of their genotype at the *SAG101* locus (Figure 9A). HR phenotype correlated with increased expression of *PR-1* gene in both *HRT SAG101* and *HRT sag101* plants (Figure 9B). However, all the *HRT sag101* plants showed susceptibility to TCV and allowed increased accumulation of TCV in the distal tissues (Figures 9C, 9D, Table S1). The susceptible phenotype correlated with a significant reduction in SA and SAG accumulation in TCV inoculated *HRT sag101* plants (Figure 9E). Together, these results suggested that SAG101 is required for HRT-mediated resistance.

To determine if the *sag101* mutation compromised resistance to TCV by affecting the accumulation of HRT, we mobilized the *HRT-FLAG* transgene into *HRT sag101* plants and analyzed HRT-FLAG levels. The *HRT sag101* plants contained wt-like levels of HRT protein, before and after TCV inoculation (Figure 9F). In addition, as in Di-17 plants, all the HRT protein was present in the membranous fraction of extracts from *HRT sag101* plants (Figure 9G). These results indicate that the inability of *HRT sag101* plants to induce pathogen-responsive SA accumulation was not due to the altered levels or localization of the R protein. Notably, pretreatment with SA or its analog BTH restored resistance in *HRT sag101* plants (Figure 9H). SA pretreatment also restored resistance in *HRT eds1*, but not in *HRT pad4* plants (Figure 9H). The resistant and susceptible phenotypes in *HRT sag101/HRT eds1* and *HRT pad4* plants, respectively, correlated with *HRT* transcript levels; BTH treatment increased *HRT* transcript levels in *HRT sag101* and *HRT eds1*, but not in *HRT pad4* plants (Figure 9I). Thus, PAD4, but not EDS1 or SAG101, is required for the SA-mediated induction of *HRT*. Together, these results suggest that SAG101 does fulfill an independent function in HRT-mediated signaling.

## Discussion

Two functionally redundant isoforms of EDS1 in the Arabidopsis genome participate in resistance signaling such that the presence of either isoform is sufficient to mediate R-derived defense against microbial pathogens. Consistent with the co-operative roles of PAD4 and SAG101 in EDS1 function, either EDS1 isoforms can interact with PAD4 and SAG101, as well as form ternary complexes with these proteins. While SAG101 can drive the nuclear localization of EDS1, the presence of PAD4 can disrupt this to retain some EDS1 in the cytosol. This raises the possibility that the relative levels of SAG101 and PAD4 may drive the subcellular localization of EDS1. Conversely, EDS1 can also drive the localization of SAG101. For example, even though the majority of the EDS1-SAG101 or the SAG101-EDS1-PAD4 complexes are present in the nucleus, preferential retention of EDS1 in the cytosol via the addition of a nuclear export signal relocates SAG101 and the ternary complex to the cytosol. Furthermore, these data suggest that dynamic changes in the levels of EDS1/PAD4/SAG101 could drive the subcellular localization of the binary/ternary complexes to regulate defense signaling. These results also offer a possible mechanistic explanation for the inability of *EDS1-NES* to fully complement *eds1* phenotypes [18].

A significantly large recovery of the EDS1-PAD4 and EDS-SAG101 complexes versus the SAG101-EDS1-PAD4 complex in co-IP studies suggests that EDS1 might be primarily present in a complex with PAD4 and SAG101. However, at this point we cannot discount the possibility that SAG101-EDS1-PAD4 complex is inherently unstable in cell free extracts. Indeed, earlier studies were unsuccessful in isolating SAG101-EDS1-PAD4 complex from cell free extracts [17], [33]. Notably, both PAD4 and SAG101 interact with the LP (1–350 aa in EDS1-90 and 1–357 aa in EDS1-80) domain of EDS1-80, which could potentially lead to steric hindrances resulting in protein instability. Intriguingly, our interaction studies with EDS1-LP and EP domains are not consistent with a previous report [16], which showed that the EP domain (351–623 aa) of EDS1-90 can self-interact, however the full-length EDS1-90 protein is required for interaction with PAD4. We find that the LP, but not the EP domains of both EDS1 isoforms, are required for interactions with self as well with PAD4 and SAG101. One possibility for these discrepancies is that our studies were carried out *in planta*, which likely better mimic the native environment than those in the Feys et al [17] study. Nonetheless, detection of the SAG101-EDS1-PAD4 complex required the full length EDS1 protein.

Unlike EDS1 and PAD4, which are well known to be essential for R protein-mediated defense to several pathogens [11], [16], [17], [20], [28], [29], SAG101 has been assigned a redundant role [17], [19]. This is because mutations in *SAG101* do not compromise RPM1, RPS4, or RPP2-mediated resistance to the respective bacterial or oomycete pathogens, but does enhance susceptibility in *pad4* plants [17]. In comparison to single mutants, simultaneous mutations in *SAG101* and *PAD4* also confer increased susceptibility to non-host pathogens [19], further supporting the functional redundancy between SAG101 and PAD4. We find that at least in the case of HRT-mediated signaling, SAG101 is as important as EDS1 and PAD4, since the absence of SAG101 alone can compromise HRT-mediated resistance to TCV. A PAD4-independent role for SAG101 is further supported by the fact that unlike *PAD4*, *SAG101* is not required for the SA-mediated induction of *HRT*. Interestingly, similar to the *eds1-1* and *pad4-1* mutations [22], the *sag101-1* mutation also reduced TCV-induced SA levels. Thus, similar to EDS1 and PAD4, SAG101 also regulates TCV-induced SA accumulation and might function in a feedback loop with SA.

The *sag101* plants also show a nominal reduction in the levels of EDS1-90 and PAD4 proteins [17], which could contribute towards susceptibility to TCV in these plants. However, this is unlikely because *sag101* plants are unaltered in RPS4-mediated resistance, which like HRT, is dependent on EDS1 and PAD4. In this regard, it is interesting to note that while loss of both EDS1 isoforms is thought to destabilize PAD4 and SAG101 [17] and thereby pathogen resistance, lack of a single isoform does not compromise resistance to TCV or *avrRps4* bacteria. Clearly, the levels of EDS1, PAD4, SAG101 essential for initiating signaling response needs further clarification. It is possible that these proteins initiate normal signaling even at very low levels. This is not unusual as *HRT cry2* and *HRT phot2* plants show normal HR even though they contain significantly lower levels of HRT compared to wild type plants [24].

An important aspect that has not been addressed thus far is whether EDS1, PAD4, and SAG101 function as individual proteins or in a complex. Clearly, at least PAD4 fulfills a unique function in HRT-mediated signaling on its own; SA-mediated increase in HRT requires PAD4, but not EDS1 or SAG101. Similarly, only EDS1 facilitated HRT+CP-mediated HR to TCV. The EDS1-80-EDS-90 complex also does not appear to be essential for HRT- or RPS4-mediated signaling, since these continue to function in the *eds1-80* or *eds1-90* mutants. Interaction between EDS1 and HRT and the fact that EDS1 forms a complex with PAD4 and SAG101 suggests that these proteins might be part of a multi-protein complex and thus regulate signaling by modulating the activity of HRT. The absence of EDS1-HRT interaction in the BiFC assay suggests that EDS1 may associate indirectly with HRT. Interestingly, EDS1 does not dissociate from HRT in the presence of CP, suggesting that CP-triggered activation of HRT does not involve the release of EDS1. Whether CP-triggered activation of HRT utilizes the EDS1, PAD4, and SAG101 proteins individually or as complexes needs further clarification. However, the requirements for all three proteins do support the notion that the SAG101-EDS1-PAD4 ternary complex might be important. It will indeed be important to establish the biochemical activities of EDS1, PAD4 and SAG101 proteins in order to accurately access the importance of the ternary complex in R protein-mediated signaling.

## Materials and Methods

### Plant growth conditions, genetic analysis and generation of transgenic plants

Plants were grown in MTPS 144 Conviron (Winnipeg, MB, Canada) walk-in-chambers at 22°C, 65% relative humidity and 14 hour photoperiod. The photon flux density of the day period was 106.9 µmoles m^−2^ s^−1^ and was measured using a digital light meter (Phytotronic Inc, Earth city, MO). All crosses were performed by emasculating the flowers of the recipient genotype and pollinatng with the pollen from the donor. F2 plants showing the wt genotype at the mutant locus were used as controls in all experiments. The wt and mutant alleles were identified by PCR, CAPS, or dCAPS analysis [21]–[26]. The *EDS1* KO mutant in At3**g48080 and At3**g48090 were isolated by screening SALK_019545 and SALK_071051 insertion lines, respectively. This *EDS1 KO* in At3**g48090 was previously designated *eds1-22* and redesignated here as *eds1-90*. The homozygous insertion lines were verified by sequencing PCR products obtained with primers specific for the T-DNA left border in combination with an *EDS1*-specific primer (Table S2).

The full length cDNA corresponding to *EDS1-80* and *EDS1-90* genes were PCR amplified using linkered primers and cloned downstream of 35S promotor in pRTL2.GUS. For Arabidopsis transformation, the fragment containing the promotor, cDNA and terminator was removed from pRTL2-EDS1 vectors and cloned into binary vector pCambia or pBAR. These clones in binary vectors were moved into *Agrobacterium tumefaciens* strain MP90 by electroporation and were used to transform Arabidopsis via the floral dip method. Selection of transformants was carried out on medium containing hygromycin or soil sprayed with the herbicide BASTA.

### RNA extraction, northern analyses and RT-PCR

Small-scale extraction of RNA from one or two leaves was performed with the TRIzol reagent (Invitrogen, CA), following the manufacturer's instructions. Northern blot analysis and synthesis of random-primed probes for *PR-1* and *PR-2* were carried out as described previously [26].

RNA quality and concentration were determined by gel electrophoresis and determination of A_260_. Reverse transcription (RT) and first strand cDNA synthesis were carried out using Superscript II (Invitrogen, CA). Two-to-three independent RNA preparations were used for RT-PCR and each of these were analyzed at least twice by RT-PCR. The RT-PCR was carried out for 35 cycles in order to determine absolute levels of transcripts. The number of amplification cycles was reduced to 21–25 in order to evaluate and quantify differences among transcript levels before they reached saturation. The amplified products were quantified using ImageQuant TL image analysis software (GE, USA). Gene-specific primers used for RT-PCR analysis are described in Table S2.

### Trypan-blue staining

The leaves were vacuum-infiltrated with trypan-blue stain prepared in 10 mL acidic phenol, 10 mL glycerol, and 20 mL sterile water with 10 mg of trypan blue. The samples were placed in a heated water bath (90°C) for 2 min and incubated at room temperature for 2–12 h. The samples were destained using chloral hydrate (25 g/10 mL sterile water; Sigma), mounted on slides and observed for cell death with a compound microscope. The samples were photographed using an AxioCam camera (Zeiss, Germany) and images were analyzed using Openlab 3.5.2 (Improvision) software.

### Pathogen infections

The bacterial strain DC3000 derivatives containing pVSP61 (empty vector), or *avrRps4* were grown overnight in King's B medium containing rifampicin and kanamycin (Sigma, MO). The bacterial cells were harvested, washed and suspended in10 mM MgCl_2_. The cells were diluted to a final density of 10^5^ CFU/mL (A_600_) and used for infiltration. The bacterial suspension was injected into the abaxial surface of the leaf using a needle-less syringae. Three leaf discs from the inoculated leaves were collected at 0 and 3 or 6 dpi. The leaf discs were homogenized in 10 mM MgCl_2_, diluted 10^3^ or 10^4^ fold and plated on King's B medium.

Transcripts synthesized *in vitro* from a cloned cDNA of TCV using T7 RNA polymerase were used for viral infections. For inoculations, the viral transcript was suspended at a concentration of 0.05 µg/ µL in inoculation buffer, and the inoculation was performed as described earlier [31]. After viral inoculations, the plants were transferred to a Conviron MTR30 reach-in chamber maintained at 22°C, 65% relative humidity and 14 hour photoperiod. HR was determined visually three-to-four days post-inoculation (dpi). Resistance and susceptibility was scored at 14 to 21 dpi and confirmed by northern gel blot analysis. Susceptible plants showed stunted growth, crinkling of leaves and drooping of the bolt.

### Conductivity assays

A protocol adapted from Dellagi et al. [34] was used for conductivity measurements.

Briefly, 5 leaf discs per plant (7 mm) were removed with a cork borer, floated in distilled water for 50 min, and subsequently transferred to tubes containing 5 ml of distilled water. Conductivity of the solution was determined with an NIST traceable digital Conductivity Meter (Fisher Scientific) at the indicated time points. Standard deviation was calculated from four replicate measurements per genotype per experiment.

### SA and SAG quantification

SA and SAG quantifications were carried out from ∼300 mg of leaf tissue as described before [23].

### Chemical treatment of plants

SA or BTH treatments were carried out by spraying or subirrigating 3-week-old plants with 500 µM SA or 100 µM BTH, respectively.

### Protein extraction and immunoblot analysis

Proteins were extracted in buffer containing 50 mM Tris-HCl, pH7.5, 10% glycerol, 150 mM NaCl, 10 mM MgCl_2_, 5 mM EDTA, 5 mM DTT, and 1 X protease inhibitor cocktail (Sigma-Aldrich, St. Louis, MO). Protein concentration was measured by the Bio-RAD protein assay (Bio-Rad, CA).

For Ponceau-S staining, PVDF membranes were incubated in Ponceau-S solution (40% methanol (v/v), 15% acetic acid (v/v), 0.25% Ponceau-S). The membranes were destained using deionized water.

For soluble versus microsomal fractionations, proteins were extracted in buffer containing 50 mM Tris-MES, pH 8.0, 0.5 M sucrose, 1 mM MgCl_2_, 10 mM EDTA, 10 mM EGTA, 10 mM ascorbic acid, 5 mM DTT and 1X protease inhibitor cocktail (Sigma-Aldrich, St. Louis, MO). Total protein extract was centrifuged at 10,000 g followed by a second centrifugation at 125,000 g. The microsomal fraction was suspended in a buffer containing 5 mM potassium phosphate pH 7.8, 2 mM DTT and 1 X protease inhibitor cocktail.

Proteins (30–50 µg) were fractionated on a 7–10% SDS-PAGE gel and subjected to immunoblot analysis using α-CP, α-MYC, α-FLAG (Sigma-Aldrich, St. Louis, MO) or α-GFP antibody. Immunoblots were developed using ECL detection kit (Roche) or alkaline-phosphatase-based color detection.

Coimmunoprecipitations were carried out as described earlier [24].

### Protoplast isolation and transfection assays

Protoplasts were isolated from three-week-old Arabidopsis Col-0 plants as described earlier [35]. For protoplast transfection, ∼10^4^ protoplasts were incubated at room temperature with 20 µg of plasmid DNA and an equal volume of solution containing 40% PEG 4000, 0.1 M CaCl_2_ and 0.2 M mannitol. After 5 min, 3 ml of wash solution containing 154 mM NaCl, 125 mM CaCl2, 5 mM KCl, 5 mM glucose, 2 mM MES (pH 5.7) was added slowly to the protoplast and the protoplasts were pelleted by centrifugation at 100 x g for 1 min. The protoplasts were washed twice and finally suspended in 1 ml of wash solution. The protoplasts were incubated in a round bottom glass vial for 12 h prior to protein extraction.

### Confocal microscopy

For confocal imaging, samples were scanned on an Olympus FV1000 microscope (Olympus America, Melvile, NY). GFP (YFP), CFP (Cerulean) and RFP were excited using 488, 440, and 543 nm laser lines, respectively. Constructs were made using pSITE [36] or pEarlyGate binary vectors using Gateway technology and introduced in *A. tumefaciens* strain LBA4404 or MP90 for agroinfiltration into *N. benthamiana* or Arabidopsis, respectively. Agrobacterium strains carrying various constructs were infiltrated into wild-type or transgenic *N. benthamiana* plants expressing CFP-tagged nuclear protein H2B or Arabidopsis plants. 48 h later, water-mounted sections of leaf tissue were examined by confocal microscopy using a water immersion PLAPO60XWLSM 2 (NA 1.0) objective on a FV1000 point-scanning/point-detection laser scanning confocal 3 microscope (Olympus) equipped with lasers spanning the spectral range of 405–633 nm. RFP, CFP and GFP overlay images (40X magnification) were acquired at a scan rate of 10 ms/pixel. Images were acquired sequentially when multiple fluors were used. Olympus FLUOVIEW 1.5 was used to control the microscope, image acquisition and the export of TIFF files.

## Supporting Information

Figure S1 **Sequence alignment and RT-PCR analysis of EDS1 isoforms.** (**A**) Amino acid alignment of EDS1-80 and EDS1-90 isoforms from Col-0 ecotype. Identical resides are shaded in blue. Sequence alignment was carried out using ClustalW in the Megalign program of the DNASTAR package. (**B**) RT-PCR analysis showing *EDS1-80* and *EDS1-90* transcript levels in Col-0 plants treated with water or BTH for 48 h before sampling. The levels of *β-tubulin* were used as a internal control to normalize the amount of cDNA template. (**C**) RT-PCR analysis showing *EDS1-80* and *EDS1-90* transcript levels in mock and TCV inoculated Di-17 plants. Plants were sampled 48 h post inoculations. The levels of *β-tubulin* were used as an internal control to normalize the amount of cDNA template. (**D**) RT-PCR analysis showing *EDS1-80* and *EDS1-90* transcript levels in Col-0, L*er* and Di-17 plants. The levels of *β-tubulin* were used as a internal control to normalize the amount of cDNA template. (**E**) Partial genomic DNA sequence alignment of EDS1-80 isoforms amplified from Col-0, L*er* and Di-17 plants. Identical resides are shaded in blue. Red box indicates region deleted in L*er* and Di-17 sequences. Sequence alignment was carried out using ClustalW in the Megalign program of the DNASTAR package. (**F**) RT-PCR analysis showing *EDS1-80* and *EDS1-90* transcript levels in Col-0 and Ws ecotypes. The level of *β-tubulin* was used as an internal control to normalize the amount of cDNA template.
(TIFF)

Figure S2 **Mutations in** ***EDS1-80*** **or** ***EDS1-90*** **do not compromise resistance to TCV. (A)** RT-PCR analysis showing *EDS1-80* and *EDS1-90* transcript levels in Col-0 and *eds1-80* plants. The levels of *β-tubulin* were used as a internal control to normalize the amount of cDNA template. (**B**) Typical morphological phenotypes of TCV inoculated Di-17, *HRT eds1-80*, *HRT eds1-90* (*HRT eds1-22*) and *HRT eds1-1* plants. R and S indicate resistant and susceptible genotypes, respectively. (**C**) Immunoblot showing levels of TCV coat protein (CP) in total proteins extracted from systemic tissues of mock- or TCV-inoculated plants. Ponceau-S staining of the Western blot was used as the loading control.
(TIFF)

Figure S3 **Mutations in** ***EDS1-80*** **or** ***EDS1-90*** **do not compromise** ***RPS4*** **-mediated resistance.** (**A**) Photograph showing phenotypes produced upon infiltration of 10^5^ colony forming units (CFU)/ml *avrRps4* bacteria. The leaves were photographed at 6 days post inoculation (dpi). (**B**) Trypan blue stained leaf showing microscopic cell death phenotype on *avrRps4* inoculated leaves. Scale bars, 270 microns. (**C**) *PR-1* gene expression in *avrRps4* inoculated plants. Leaves were sampled at 2 dpi. Ethidium bromide staining of rRNA was used as a loading control. (**D**) Salicylic acid (SA) and SA glucoside (SAG) levels in indicated genotypes at 0 and 48 h post inoculation with *avrRps4*. Asterisks indicate data statistically significant from wt Col-0 ecotype (P<0.05, n = 3). The error bars indicate SD. (**E**) Growth of *avrRps4* bacteria on indicated genotypes. The error bars indicate SD. Asterisks indicate data statistically significant from wt (Col-0, P<0.05 n = 4).
(TIFF)

Figure S4 **Transcript levels and cell death phenotype in** ***eds1-1*** **plants overexpressing** ***EDS1-80*** **or** ***EDS1-90*** **.** (**A–B**) Expression of *EDS1-80* (**A**) and *EDS1-90* (**B**) in *eds1-1* and three independent T2 transgenic plants overexpressing *EDS1-80* or *EDS1-90* genes in *eds1-1* background. Total RNA was extracted from 4-week-old plants and ethidium bromide staining of rRNA was used as the loading control. (**C**) Trypan blue stained leaf showing microscopic cell death phenotype in *avrRps4* inoculated leaves. Scale bar, 270 microns.
(TIFF)

Figure S5 **Coimmunoprecipitation assays showing interaction between HRT and EDS1.** (**A**) Co-immunoprecipitation (IP) of HRT with EDS1-90-FLAG expressed under their native promoters. *N. benthamiana* plants were agroinfiltrated and total extracts (input) and immunoprecipitated proteins were analyzed with α-MYC and α-FLAG. (**B**) IP of HRT-MYC with EDS1-90-FLAG in Arabidopsis. The Arabidopsis protoplasts prepared from Col-0 plants were transfected with constructs expressing HRT-MYC and EDS1-90-FLAG and total extracts (input) and immunoprecipitated proteins were analyzed with α-MYC and α-FLAG. (**C**–**D**) Levels of EDS1 (**C**) and HRT (**D**) proteins expressed under either the 35S or their native promoters. *N. benthamiana* were agroinfiltrated and total extracts were analyzed with α-MYC or α-FLAG.
(TIFF)

Figure S6 **Coimmunoprecipitation and BiFC assays showing interaction of EDS1-90 to itself and to SAG101 and PAD4 proteins.** (**A**) IP of EDS1-90-MYC protein with EDS1-90-FLAG. *N. benthamiana* plants were agroinfiltrated and total extracts (input) and immunoprecipitated proteins were analyzed with α-MYC and α-FLAG. (**B**) Co-IP of EDS1-90-MYC and PAD4-MYC with EDS1-80-FLAG. *N. benthamiana* plants were agroinfiltrated and total extracts (input) and immunoprecipitated proteins were analyzed with α-MYC. (**C**) Confocal micrographs showing BiFC for SAG101 and PAD4 with EDS1-90 LP (lipase, 1–350 aa) and EP (EDS1-PAD4, 351–623) domains. Agroinfiltration was used to express protein in transgenic *N. benthamiana* plants expressing the nuclear marker CFP-H2B, Scale bar, 10 µM.
(TIFF)

Figure S7 **Interaction assays and localization of EDS1.** (**A**) Confocal micrographs showing localization of indicated proteins co-expressed in pairs (Scale bar, 10 µM). (**B**) Immunoblot showing levels of SAG101-MYC in *N. benthamiana* plants coexpressing EDS1-80/90-GFP and SAG101-MYC (shown in **A**). Ponceau-S staining of the Western blot was used as the loading control. (**C**) Confocal micrograph showing localization of indicated proteins co-expressed together (Scale bar, 10 µM). (**D–E**) Levels of PAD4 (**D**) and SAG101 (**E) proteins** expressed under either the 35S or their native promoters. *N. benthamiana* were agroinfiltrated and total extracts were analyzed with α-MYC. (**F**) Confocal micrographs showing localization of indicated proteins co-expressed in pairs (Scale bar, 10 µM).
(TIFF)

Figure S8 **EDS1-90 forms extranuclear complexes with PAD4 and SAG101.** Confocal micrographs showing BiFC for indicated proteins. Agroinfiltration was used to express proteins in transgenic *N. benthamiana* plants expressing the nuclear marker CFP-H2B (Scale bar, 10 µM).
(TIFF)

Figure S9 **Immunoblot showing levels of various MYC tagged EDS1 derivatives corresponding to confocal data shown in Figure 8A.** Ponceau-S staining of the Western blot was used as the loading control.
(TIFF)

Table S1 **Epistatic analysis of F2 population derived from crosses between Di-17 and various wild-type or mutant lines.**
(DOCX)

Table S2 **Primer sequences used for overexpression, knock-out analysis, BiFC and localization.**
(DOCX)
