# Supplementary figures and images for: SAG101 Forms a Ternary Complex with EDS1 and PAD4 and Is Required for Resistance Signaling against Turnip Crinkle Virus
Source: PLoS Pathog. 2011 Nov 3;7(11):e1002318. doi: 10.1371/journal.ppat.1002318 (PMC3207898; doi:10.1371/journal.ppat.1002318)

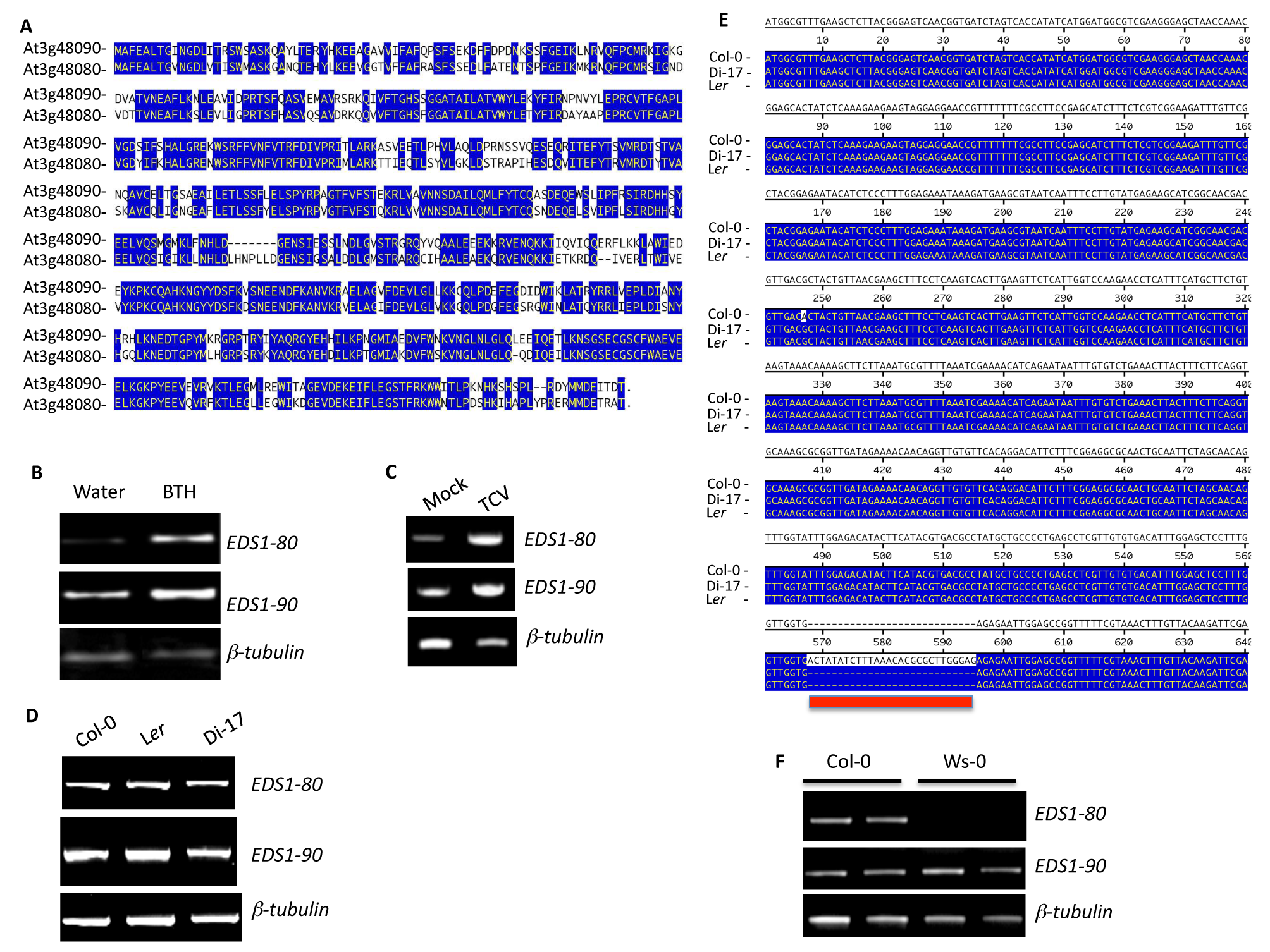

Supplement: Figure S1 — Sequence alignment and RT-PCR analysis of EDS1 isoforms. (A) Amino acid alignment of EDS1-80 and EDS1-90 isoforms from Col-0 ecotype. Identical resides are shaded in blue. Sequence alignment was carried out using ClustalW in the Megalign program of the DNASTAR package. (B) RT-PCR analysis showing EDS1-80 and EDS1-90 transcript levels in Col-0 plants treated with water or BTH for 48 h before sampling. The levels of β-tubulin were used as a internal control to normalize the amount of cDNA template. (C) RT-PCR analysis showing EDS1-80 and EDS1-90 transcript levels in mock and TCV inoculated Di-17 plants. Plants were sampled 48 h post inoculations. The levels of β-tubulin were used as an internal control to normalize the amount of cDNA template. (D) RT-PCR analysis showing EDS1-80 and EDS1-90 transcript levels in Col-0, Ler and Di-17 plants. The levels of β-tubulin were used as a internal control to normalize the amount of cDNA template. (E) Partial genomic DNA sequence alignment of EDS1-80 isoforms amplified from Col-0, Ler and Di-17 plants. Identical resides are shaded in blue. Red box indicates region deleted in Ler and Di-17 sequences. Sequence alignment was carried out using ClustalW in the Megalign program of the DNASTAR package. (F) RT-PCR analysis showing EDS1-80 and EDS1-90 transcript levels in Col-0 and Ws ecotypes. The level of β-tubulin was used as an internal control to normalize the amount of cDNA template. (TIFF) [file ppat.1002318.s001.tiff]

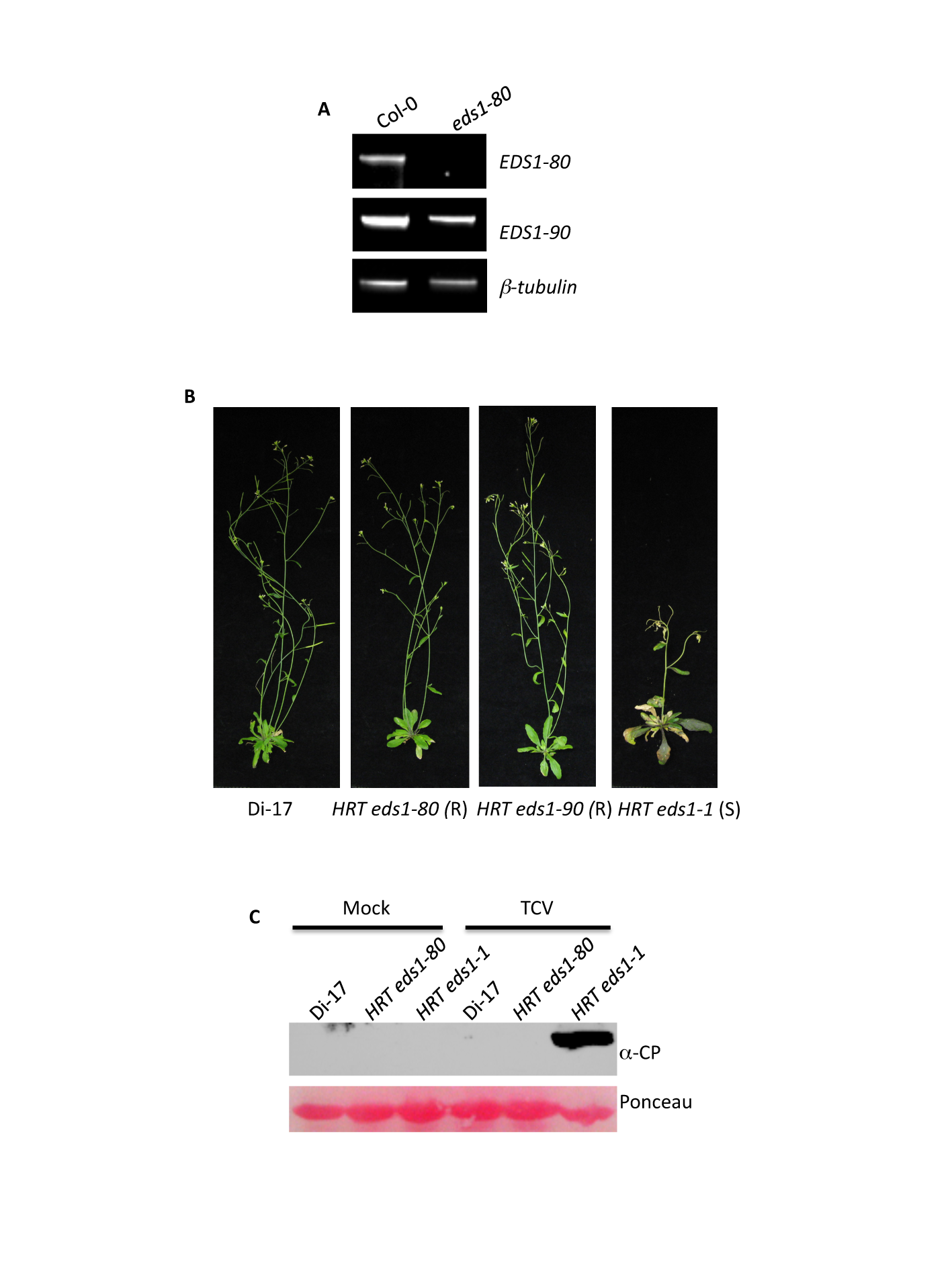

Supplement: Figure S2 — Mutations in EDS1-80 or EDS1-90 do not compromise resistance to TCV. (A) RT-PCR analysis showing EDS1-80 and EDS1-90 transcript levels in Col-0 and eds1-80 plants. The levels of β-tubulin were used as a internal control to normalize the amount of cDNA template. (B) Typical morphological phenotypes of TCV inoculated Di-17, HRT eds1-80, HRT eds1-90 (HRT eds1-22) and HRT eds1-1 plants. R and S indicate resistant and susceptible genotypes, respectively. (C) Immunoblot showing levels of TCV coat protein (CP) in total proteins extracted from systemic tissues of mock- or TCV-inoculated plants. Ponceau-S staining of the Western blot was used as the loading control. (TIFF) [file ppat.1002318.s002.tiff]

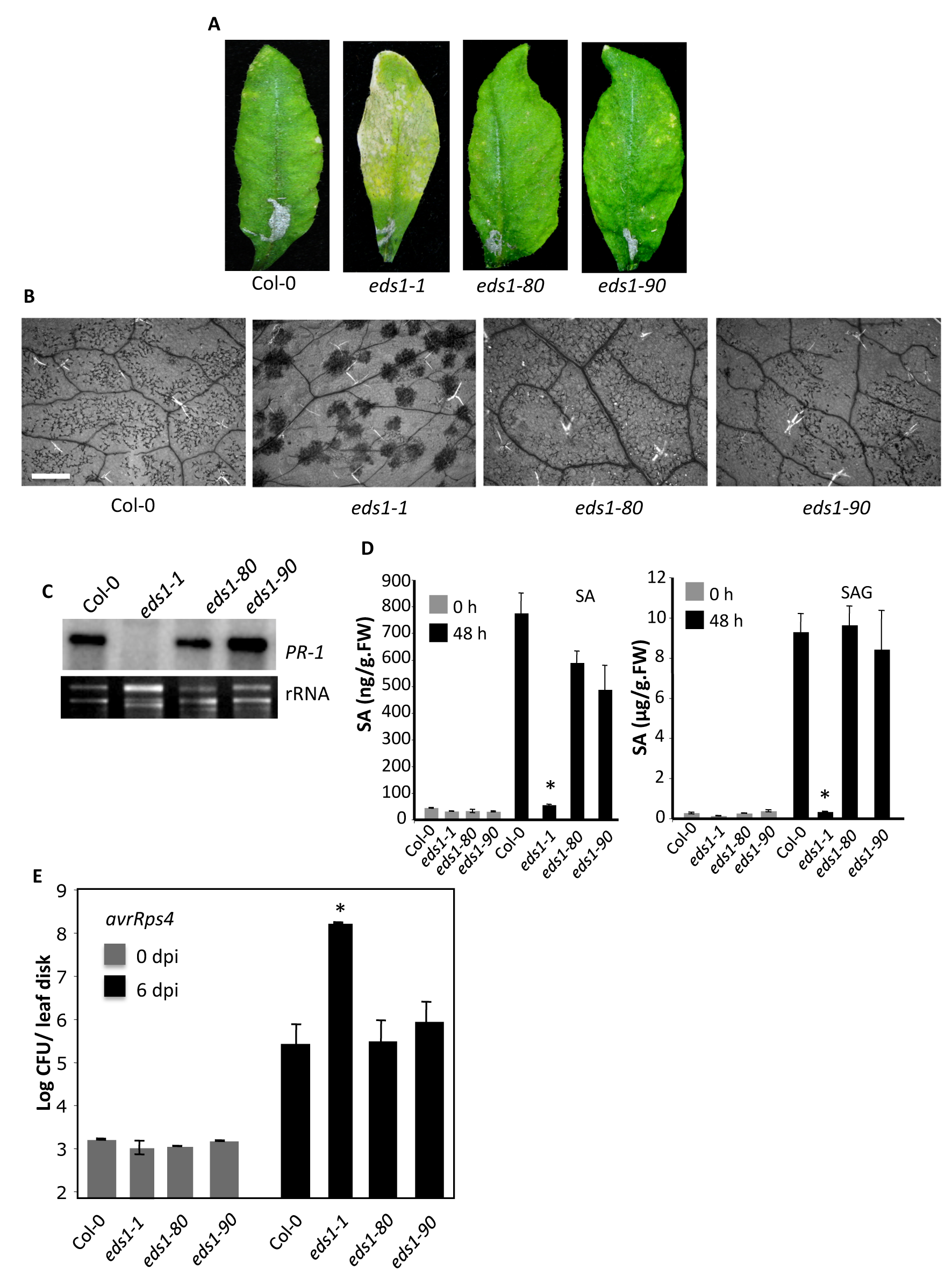

Supplement: Figure S3 — Mutations in EDS1-80 or EDS1-90 do not compromise RPS4 -mediated resistance. (A) Photograph showing phenotypes produced upon infiltration of 105 colony forming units (CFU)/ml avrRps4 bacteria. The leaves were photographed at 6 days post inoculation (dpi). (B) Trypan blue stained leaf showing microscopic cell death phenotype on avrRps4 inoculated leaves. Scale bars, 270 microns. (C) PR-1 gene expression in avrRps4 inoculated plants. Leaves were sampled at 2 dpi. Ethidium bromide staining of rRNA was used as a loading control. (D) Salicylic acid (SA) and SA glucoside (SAG) levels in indicated genotypes at 0 and 48 h post inoculation with avrRps4. Asterisks indicate data statistically significant from wt Col-0 ecotype (P<0.05, n = 3). The error bars indicate SD. (E) Growth of avrRps4 bacteria on indicated genotypes. The error bars indicate SD. Asterisks indicate data statistically significant from wt (Col-0, P<0.05 n = 4). (TIFF) [file ppat.1002318.s003.tiff]

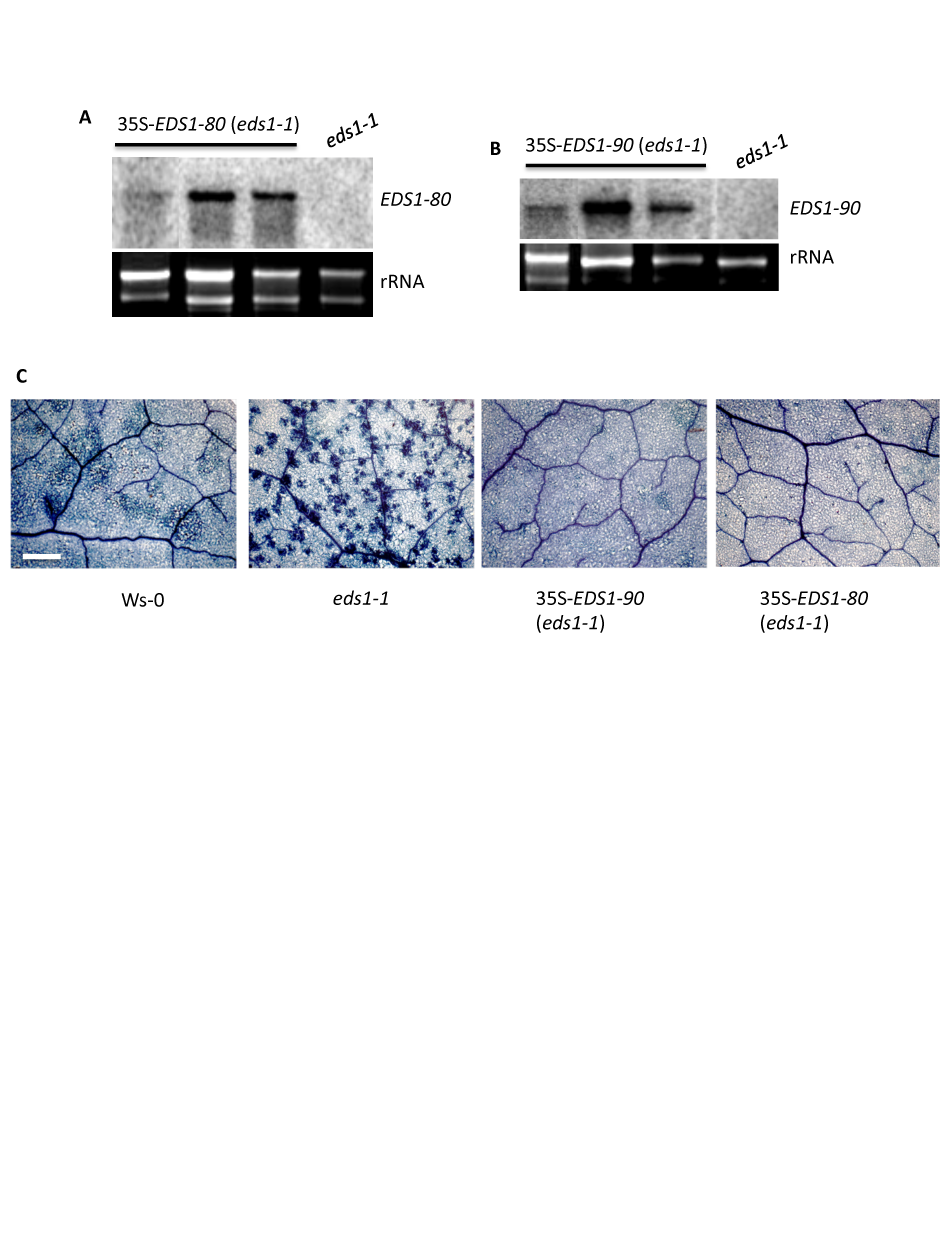

Supplement: Figure S4 — Transcript levels and cell death phenotype in eds1-1 plants overexpressing EDS1-80 or EDS1-90 . (A–B) Expression of EDS1-80 (A) and EDS1-90 (B) in eds1-1 and three independent T2 transgenic plants overexpressing EDS1-80 or EDS1-90 genes in eds1-1 background. Total RNA was extracted from 4-week-old plants and ethidium bromide staining of rRNA was used as the loading control. (C) Trypan blue stained leaf showing microscopic cell death phenotype in avrRps4 inoculated leaves. Scale bar, 270 microns. (TIFF) [file ppat.1002318.s004.tiff]

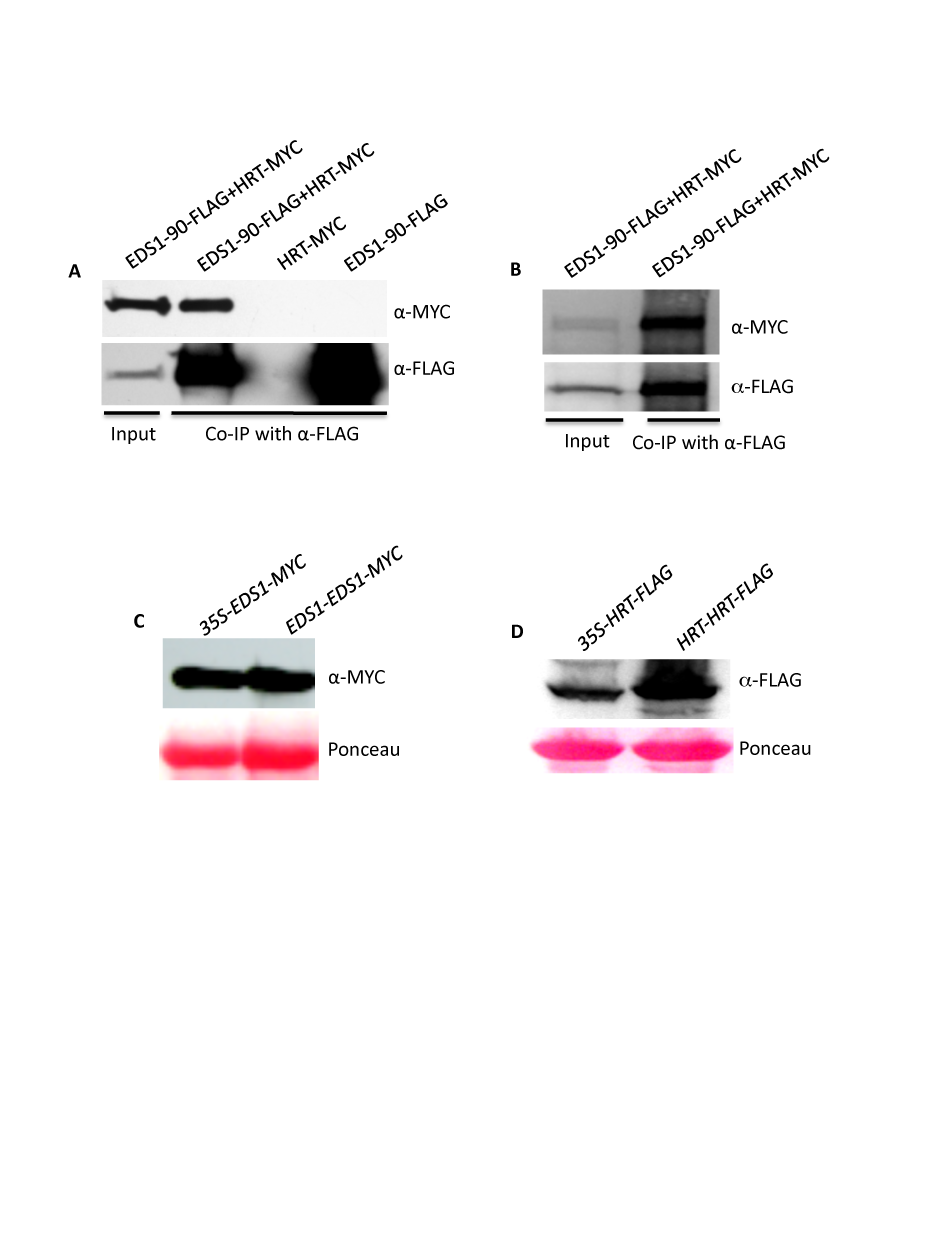

Supplement: Figure S5 — Coimmunoprecipitation assays showing interaction between HRT and EDS1. (A) Co-immunoprecipitation (IP) of HRT with EDS1-90-FLAG expressed under their native promoters. N. benthamiana plants were agroinfiltrated and total extracts (input) and immunoprecipitated proteins were analyzed with α-MYC and α-FLAG. (B) IP of HRT-MYC with EDS1-90-FLAG in Arabidopsis. The Arabidopsis protoplasts prepared from Col-0 plants were transfected with constructs expressing HRT-MYC and EDS1-90-FLAG and total extracts (input) and immunoprecipitated proteins were analyzed with α-MYC and α-FLAG. (C–D) Levels of EDS1 (C) and HRT (D) proteins expressed under either the 35S or their native promoters. N. benthamiana were agroinfiltrated and total extracts were analyzed with α-MYC or α-FLAG. (TIFF) [file ppat.1002318.s005.tiff]

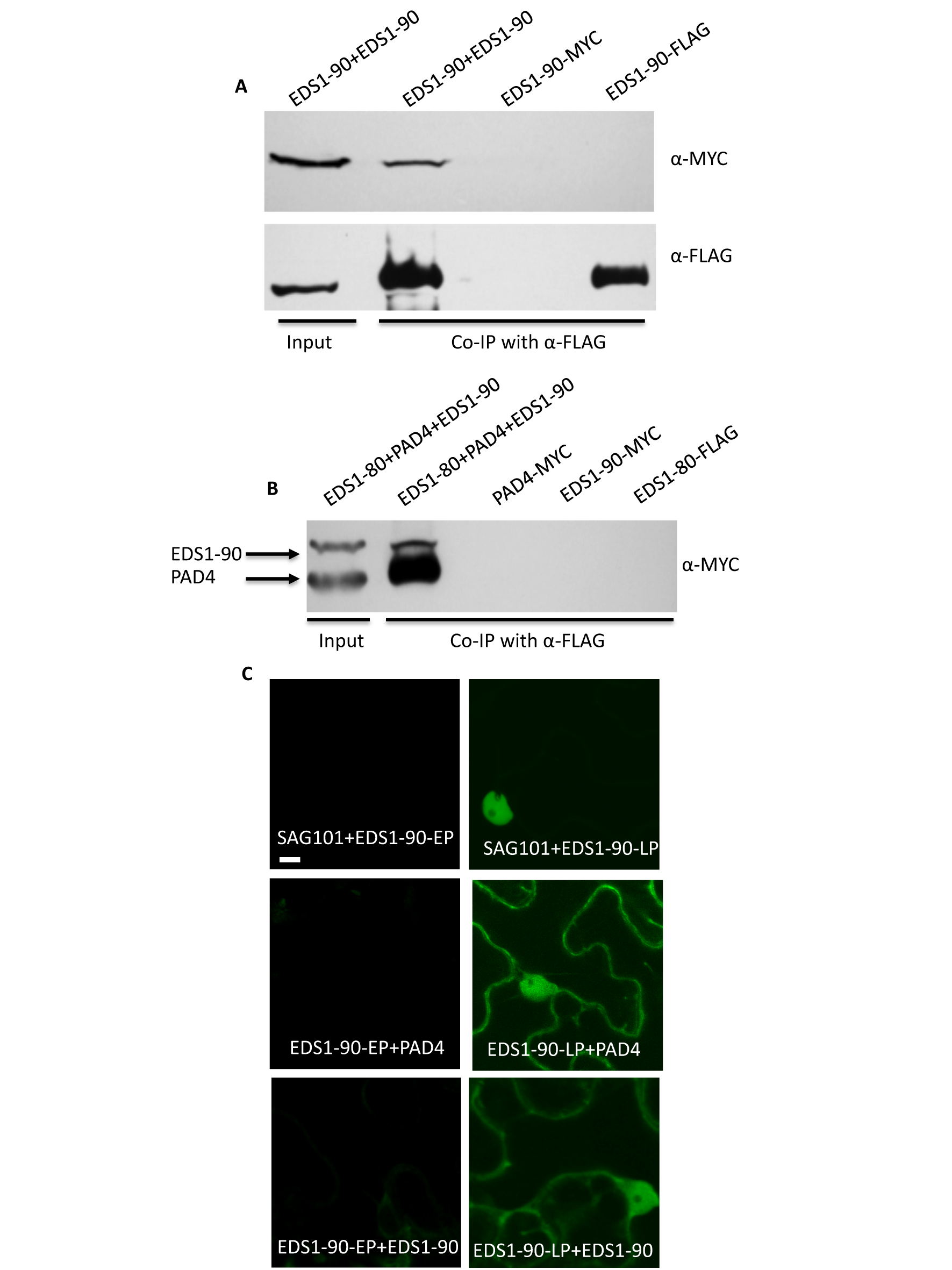

Supplement: Figure S6 — Coimmunoprecipitation and BiFC assays showing interaction of EDS1-90 to itself and to SAG101 and PAD4 proteins. (A) IP of EDS1-90-MYC protein with EDS1-90-FLAG. N. benthamiana plants were agroinfiltrated and total extracts (input) and immunoprecipitated proteins were analyzed with α-MYC and α-FLAG. (B) Co-IP of EDS1-90-MYC and PAD4-MYC with EDS1-80-FLAG. N. benthamiana plants were agroinfiltrated and total extracts (input) and immunoprecipitated proteins were analyzed with α-MYC. (C) Confocal micrographs showing BiFC for SAG101 and PAD4 with EDS1-90 LP (lipase, 1–350 aa) and EP (EDS1-PAD4, 351–623) domains. Agroinfiltration was used to express protein in transgenic N. benthamiana plants expressing the nuclear marker CFP-H2B, Scale bar, 10 µM. (TIFF) [file ppat.1002318.s006.tiff]

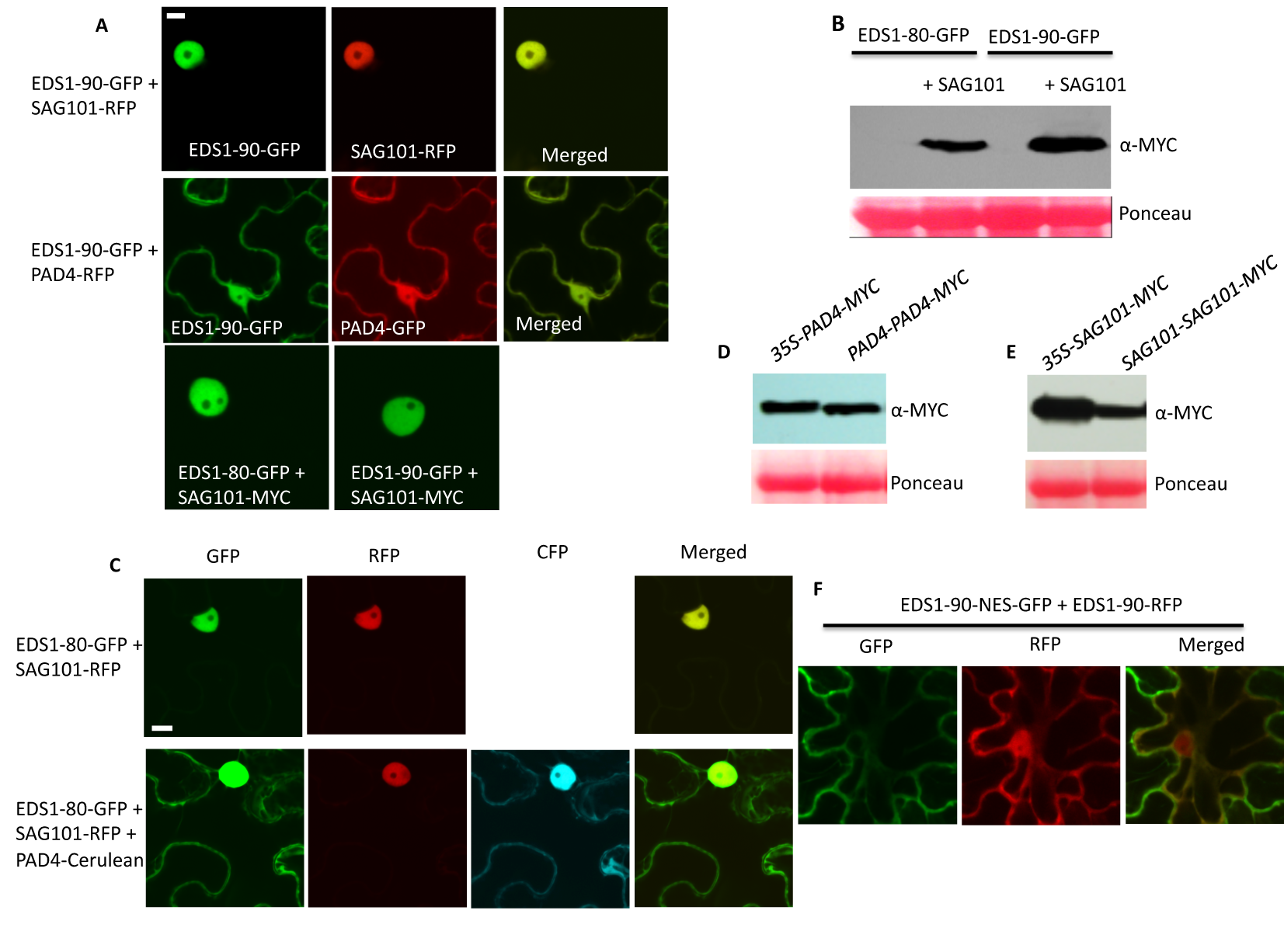

Supplement: Figure S7 — Interaction assays and localization of EDS1. (A) Confocal micrographs showing localization of indicated proteins co-expressed in pairs (Scale bar, 10 µM). (B) Immunoblot showing levels of SAG101-MYC in N. benthamiana plants coexpressing EDS1-80/90-GFP and SAG101-MYC (shown in A). Ponceau-S staining of the Western blot was used as the loading control. (C) Confocal micrograph showing localization of indicated proteins co-expressed together (Scale bar, 10 µM). (D–E) Levels of PAD4 (D) and SAG101 (E) proteins expressed under either the 35S or their native promoters. N. benthamiana were agroinfiltrated and total extracts were analyzed with α-MYC. (F) Confocal micrographs showing localization of indicated proteins co-expressed in pairs (Scale bar, 10 µM). (TIFF) [file ppat.1002318.s007.tiff]

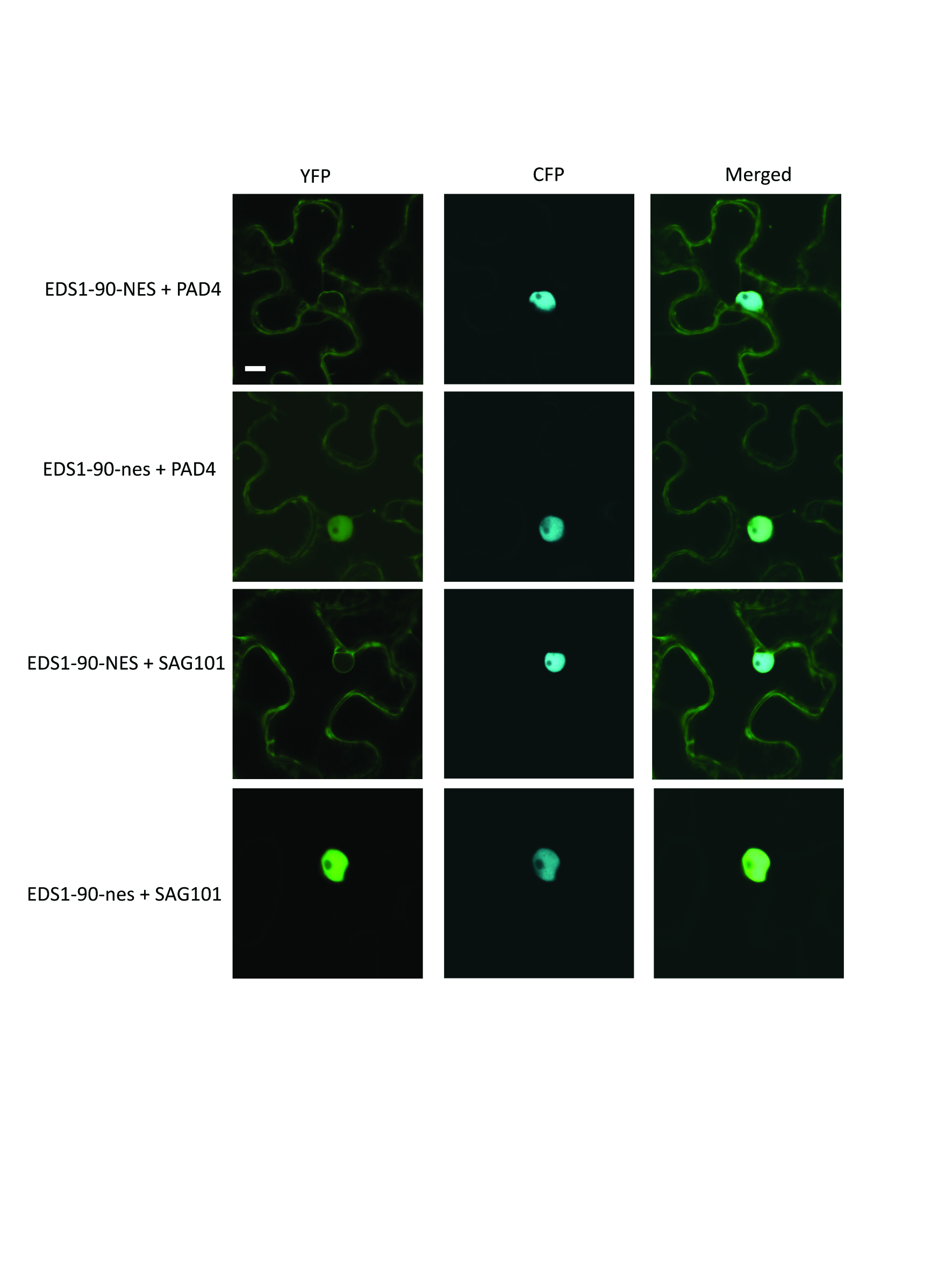

Supplement: Figure S8 — EDS1-90 forms extranuclear complexes with PAD4 and SAG101. Confocal micrographs showing BiFC for indicated proteins. Agroinfiltration was used to express proteins in transgenic N. benthamiana plants expressing the nuclear marker CFP-H2B (Scale bar, 10 µM). (TIFF) [file ppat.1002318.s008.tiff]

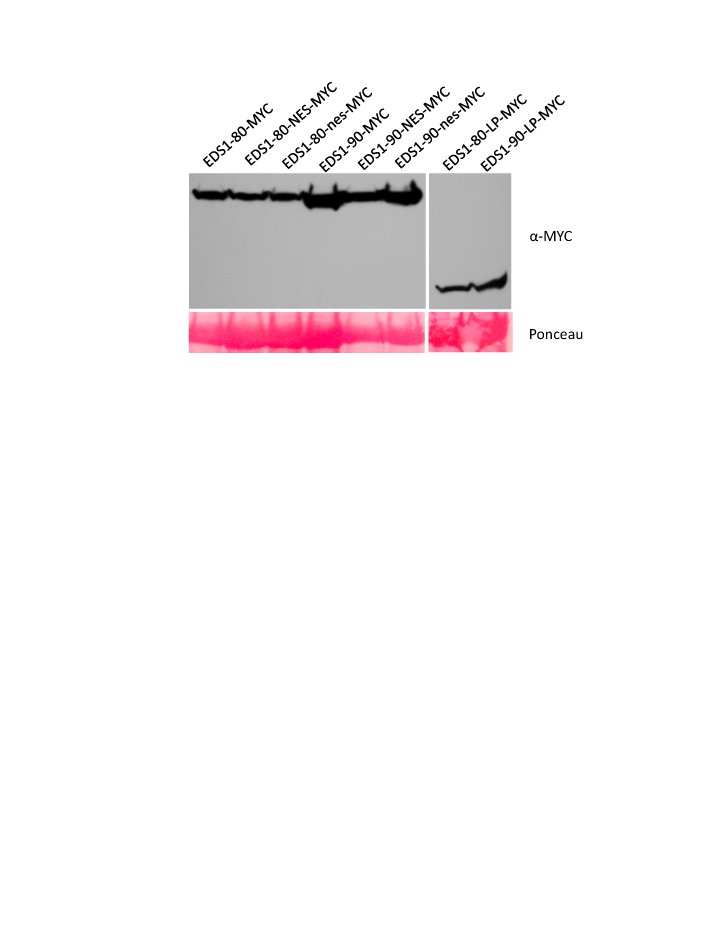

Supplement: Figure S9 — Immunoblot showing levels of various MYC tagged EDS1 derivatives corresponding to confocal data shown in Figure 8A. Ponceau-S staining of the Western blot was used as the loading control. (TIFF) [file ppat.1002318.s009.tiff]
