# Supplementary material for: SAG101 Forms a Ternary Complex with EDS1 and PAD4 and Is Required for Resistance Signaling against Turnip Crinkle Virus
Source: PLoS Pathog. 2011 Nov 3;7(11):e1002318. doi: 10.1371/journal.ppat.1002318 (PMC3207898; doi:10.1371/journal.ppat.1002318)
Supplement: Table S1 — Epistatic analysis of F2 population derived from crosses between Di-17 and various wild-type or mutant lines. (DOCX) [file ppat.1002318.s010.docx]

**Table S1.** Epistatic analysis of F2 population derived from crosses between Di-17 and various wild-type or mutant lines.

| **Cross** | **Plants analyzed** | **Genotype ^a^** | **Plants obtained** | **HR ^b^** | **R ^c^** | **S ^d^** | **χ2** ^e^ | P |
| --- | --- | --- | --- | --- | --- | --- | --- | --- |
| Di-17 x Col-0 | 165 | *HRT/-* | 97 | + | 22 | 75 | 0.58 | 0.44 |
| Di-17 x  *sag101* | 390 | *HRT/- sag101* *HRT/- SAG101/-* | 42  223 | +  + | 0  51 | 42  172 | 14.0  0.54 | <0.0002 ^f^  0.46 |
| Di-17 x  *eds1-80* | 198 | *HRT/- eds1-80**HRT/- EDS1-80/-* | 34  108 | +  + | 9  27 | 25  81 | 0.039  0.0 | 0.84  1.0 |
| Di-17 x  *eds1-90* | 185 | *HRT/- eds1-90**HRT/- EDS1-90/-* | 31  97 | +  + | 8  23 | 23  74 | 0.011  0.08 | 0.91  0.76 |
| Di-17 x  *eds1-1* ^g^ | 349 | *HRT/- eds1-1* | 60 | + | 0 | 60 | 20.0 | <0.0001 ^f^ |

^a^ The genotype at *HRT* and various mutant loci was determined by CAPS analysis

^b^ HR, hypersensitive response

^c^ Resistant

^d^ Susceptible

^e^ One degree of freedom; based on segregation of 3 susceptible:1 resistant plants

^f^ Statistically significant

^g^  From, Chandra-Shekara et al., 2004.
