# Supplementary material for: SAG101 Forms a Ternary Complex with EDS1 and PAD4 and Is Required for Resistance Signaling against Turnip Crinkle Virus
Source: PLoS Pathog. 2011 Nov 3;7(11):e1002318. doi: 10.1371/journal.ppat.1002318 (PMC3207898; doi:10.1371/journal.ppat.1002318)
Supplement: Table S2 — Primer sequences used for overexpression, knock-out analysis, BiFC and localization. (DOCX) [file ppat.1002318.s011.docx]

**Table S2.** Primer sequences used for overexpression, knock-out analysis, BiFC and localization.

| Purpose | Primers |
| --- | --- |
| Overexpresion of AT3G48080 (EDS1) | TACCTCGAGATGGCGTTTGAAGCTCTTACG  CGTTCTAGATCAGGTAGCTCTTGTTTCATCCATCATACGC |
| EDS1-80-90 attB | AAAAAGCAGGCTTAATGGCGTTTGAAGCTCTTAC (common Fwd primer) AGAAAGCTGGGTATCAGGTAGCTCTTGTTTCATC (EDS1-80) AGAAAGCTGGGTATCAGGTATCTGTTATTTCATC (EDS1-90) |
| EDS1-80-NES attB | AGAAAGCTGGGTATCAGATATCAAGTCCAGCAAGCTTAAGAGCAAGGGTAGCTCTT  GTTTCATC |
| EDS1-80-nes attB | AGAAAGCTGGGTATCAAGCATCAGCTCCAGCAGCCTTAAGAGCAAGGGTAGCTCTT  GTTTCATC |
| EDS1-90-NES attB | AGAAAGCTGGGTATCAGATATCAAGTCCAGCAAGCTTAAGAGCAAGGGTATCTGTTA  TTTCATC |
| EDS1-80-EP attB | AAAAAGCAGGCTTAAGCACAAGAGCCAGACAGTG |
| EDS1-80 LP attB | AGAAAGCTGGGTATCACATTCCAAGGTCGTCGAGAGC |
| EDS1-90-EP attB | AAAAAGCAGGCTTAAGCACAAGAGGCAGACAGTAC |
| EDS1-90-LP attB | AGAAAGCTGGGTATCACACTCCAAGGTCATTGAGCGT |
| EDS1-90-nes attB | AGAAAGCTGGGTATCAAGCATCAGCTCCAGCAGCCTTAAGAGCAAGGGTATCTGTTA  TTTCATC |
| PAD4-attB | AAAAAGCAGGCTTAATGGACGATTGTCGATTCGAG   AGAAAGCTGGGTACTAAGTCTCCATTGCGTCACT |
| PAD4-NES attB | AGAAAGCTGGGTATCAGATATCAAGTCCAGCAAGCTTAAGAGCAAGAGTCTCCATTG  CGTCACT |
| PAD4-nes attB | AGAAAGCTGGGTATCAAGCATCAGCTCCAGCAGCCTTAAGAGCAAGAGTCTCCATTG  CGTCACT |
| SAG101-attB | AAAAAGCAGGCTTAATGGAGTCTTCTTCTTCACTA   AGAAAGCTGGGTATTATTGTGACTTACCATAACTC |
| SAG101-NES attB | AGAAAGCTGGGTATCAGATATCAAGTCCAGCAAGCTTAAGAGCAAGTTGTGACTTAC  CATAACTC |
| SAG101-nes attB | AGAAAGCTGGGTATCAAGCATCAGCTCCAGCAGCCTTAAGAGCAAGTTGTGACTTAC  CATAACTC |
| HRT attB | AAAAAGCAGGCTTAATGGCTGAAGCATTTGTGTCGT  AGAAAGCTGGGTACTACTGGTCACAGTTGATAAAT |
| EDS1-80 KO screen  (SALK-019545) | TTGGATCTGCATAATCCGCT   ATCATACGCTCACGTGGATA |
| *sag101* genotyping | CACGCGTCCGAAGATCTTGGAGATAC (Fwd) ACTTCCGGGTGTTCATAAACTCGGTC (Rev) GGTGCAGCAAAACCCACACTTTTACT (dspm11)  Fwd+Rev= ~1.3kb in wt, no amplicon from mutant.  Fwd+dspm11= ~1 kb in mutant, no amplicon in wt. |
| *β-tubulin* | CGTGGATCACAGCAATACAGAGCC  CCTCCTGCACTTCCACTTCGTCTTC |
